# Predicting response to immunotherapy in gastric cancer via assessing perineural invasion-mediated inflammation in tumor microenvironment

**DOI:** 10.1186/s13046-023-02730-0

**Published:** 2023-08-11

**Authors:** Xunjun Li, Yiyun Wang, ZhongYa Zhai, Qingyi Mao, Dianjie Chen, Luxi Xiao, Shuai Xu, Qilin Wu, Keming Chen, Qiantong Hou, Qinglie He, Yuyang Shen, Manchun Yang, Zishan Peng, Siqing He, Xuanhui Zhou, Haoyang Tan, Shengwei Luo, Chuanfa Fang, Guoxin Li, Tao Chen

**Affiliations:** 1 Department of General Surgery, Nanfang Hospital, Southern Medical University, Guangdong Provincial Engineering Technology Research Center of Minimally Invasive Surgery, No. 1838, North Guangzhou Avenue, Guangzhou, 510515 Guangdong Province China; 2 The Second School of Clinical Medicine, Southern Medical University, Guangzhou, 510515 Guangdong Province China; 3 Medical Image Center, Nanfang Hospital, Southern Medical University, Guangzhou, 510515 Guangdong Province China; 4 School of Public Health, Southern Medical University, Guangzhou, 510515 Guangdong Province China; 5 Department of Gastrointestinal and Hernia Surgery, Ganzhou Hospital-Nanfang Hospital, Southern Medical University, Ganzhou, 341000 Jiangxi China

**Keywords:** Perinueral invasion(PNI), Gastric cancer(GC), Inflammatory, Tumor microenvironment(TME), Neuroinflammation infiltration(NII) score system

## Abstract

**Background:**

The perineural invasion (PNI)-mediated inflammation of the tumor microenvironment (TME) varies among gastric cancer (GC) patients and exhibits a close relationship with prognosis and immunotherapy. Assessing the neuroinflammation of TME is important in predicting the response to immunotherapy in GC patients.

**Methods:**

Fifteen independent cohorts were enrolled in this study. An inflammatory score was developed and validated in GC. Based on PNI-related prognostic inflammatory signatures, patients were divided into Clusters A and B using unsupervised clustering. The characteristics of clusters and the potential regulatory mechanism of key genes were verified by RT-PCR, western-blot, immunohistochemistry and immunofluorescence in cell and tumor tissue samples.The neuroinflammation infiltration (NII) scoring system was developed based on principal component analysis (PCA) and visualized in a nomogram together with other clinical characteristics.

**Results:**

Inflammatory scores were higher in GC patients with PNI compared with those without PNI (*P* < 0.001). NII.clusterB patients with PNI had abundant immune cell infiltration in the TME but worse prognosis compared with patients in the NII.clusterA patients with PNI and non-PNI subgroups. Higher immune checkpoint expression was noted in NII.clusterB-PNI. VCAM1 is a specific signature of NII.clusterB-PNI, which regulates PD-L1 expression by affecting the phosphorylation of STAT3 in GC cells. Patients with PNI and high NII scores may benefit from immunotherapy. Patients with low nomogram scores had a better prognosis than those with high nomogram scores.

**Conclusions:**

Inflammation mediated by PNI is one of the results of tumor-nerve crosstalk, but its impact on the tumor immune microenvironment is complex. Assessing the inflammation features of PNI is a potential method in predicting the response of immunotherapy effectively.

**Supplementary Information:**

The online version contains supplementary material available at 10.1186/s13046-023-02730-0.

## Introduction

Perineural invasion (PNI) is a common pathological phenomenon in malignant solid tumors, such as pancreatic cancer [1], gastric cancer(GC) [2], and prostate cancer [3]. PNI was first reported by Batsakis in 1985 [4], and the definition of PNI has been refined to become more accurate over the past few decades. Its clinical significance has been confirmed in multiple clinical cohort studies, and it is considered a prognostic risk factor [5]. Therefore, in some clinical tumor diagnosis and treatment guidelines [6, 7], nerve invasion is also listed as one of the indicators of adjuvant chemotherapy in addition to surgical resection in the early stage of cancer.

Although the poor prognosis of cancer patients with PNI has been recognized, the specific molecular mechanisms remain unclear. In previous studies, many researchers believed that peripheral nerve injury would cause inflammation around the nerve [8–10], triggering a series of physiological reactions, such as pain, fever and promoting tissue repair. Therefore, in the tumor microenvironment(TME), does tumor invasion of the nerve also trigger a similar neuroinflammatory response? It is important to note that a large number of studies have described a close relationship between chronic inflammation and tumor progression, especially in the study of gastrointestinal cancer [11–13].

In addition, some studies [14–16] have noted that the local inflammatory response is inevitably accompanied by an abundant response and recruitment of immune cells. Different levels of inflammatory response patterns trigger different immune responses. It has been reported in several tumor studies that nerve invasion is closely related to the inflammatory response [5, 17, 18]. It has also been suggested that nerve invasion mediates immune escape in tumor formation [19, 20]. These findings suggest a close relationship among PNI, inflammatory and immune responses.

In our study, we demonstrated that PNI was closely related to inflammatory responses in the TME and that both were associated with poor patient outcomes. Then, we identified two neuroinflammatory subtypes in GC patients with PNI and analyzed their RNA expression, somatic mutations and DNA methylation features in the TME. Based on the difference between the two subtypes, we developed an neuroinflammation infiltration(NII) scoring system for GC patients and visualized it in a nomogram. Furthermore, we assessed the clinical application prospects of this system to predict the response to immunotherapy in GC patients.

## Methods

### Data retrieval and preprocessing

A total of 15 independent cohorts, which included 1623 GC patients, 1175 colorectal cancer(CRC) patients, 413 liver cancer patients and 259 pancreatic cancer patients, were enrolled in this study(GC:TCGA-STAD, GSE62254,GSE15459,GSE84437,GSE13861,GSE26899,GSE26901,Nanfang cohort 1 and Nanfang cohort 2;CRC:TCGA-COAD,GSE39582,GSE17536;liver cancer:TCGA-LIHC;pancreatic cancer:TCGA-PAAD,GSE85916). More details of cohorts for training and validating were showed in Supplementary file 1.

Patients enrolled in this study met the following inclusion criteria: a) complete follow-up information; b) the survival time of surviving patients must be more than 100 days, and all patients who died were included; additional criteria c) clear PNI information(Applied in training cohort, TCGA-CRC cohort, Nanfang cohort1 and 2, for validating the prognostic value of PNI and constructing NII cluster system.). Overall, the information of all the datasets enrolled in this study is summarized in Supplementary Table S1.

### Assessment of prognostic value of PNI in gastric cancer

To predict the prognosis of GC patients with PNI, Kaplan‒Meier analysis was applied to reveal the overall survival difference between PNI and non-PNI patients in the training cohort as well as Nanfang cohort 1. Multivariate Cox regression analysis was used to validate the independent prognostic ability of PNI. In addition, Gene Ontology (GO) enrichment analysis and Gene Set Enrichment Analysis(GSEA) were used to investigate the differences in signal transduction pathways between the PNI and non-PNI groups.

### Development and pangastrointestinal neoplasm validation of the Prognostic Inflammatory Response-Related Gene Signature in STAD

Table S2 provides 200 inflammatory response-related genes retrieved from the Molecular Signatures database(MSigDB). The details about the calculation of inflammation score were described in Supplementary file 1.

For each patient, an inflammation score was generated using the formula below:

$$\mathrm{Inflammation\ score}\hspace{0.17em}=\hspace{0.17em}\sum\mathrm\beta\mathrm i\ast\mathrm{Expi}$$

Gene expression value Expi represents the coefficient for each gene in the final Cox model.

### Inference of infiltrating cells in the TME

Gene expression data were employed to characterize the tumor immune microenvironment of samples using a variety of bioinformatics tools. A set of markers for the TME infiltration immune cell type was derived from Bindea et al. [21]. More details were showed in Supplementary file 1.

### Identification and consensus clustering of neuroinflammatory genes for GC

To further explore the inflammatory differences within PNI, a more precise classification, or consensus clustering, was performed. Univariate Cox analysis was applied to identify genes associated with both inflammation and prognosis in patients with perineural invasion(26 genes in total). Detailed analysis procedures are provided in Supplementary file 1.

### Transcriptome analysis among PNI-related subtypes

Signal transduction pathways were investigated using GO enrichment analysis, Kyoto Encyclopedia of Genes and Genomes (KEGG) analysis, and GSEA.Then, tumor-infiltrating immune cell differences among the three subtypes were assessed using ssGSEA and the Estimate algorithm. In addition, we curated a set of gene sets based on Mariathasan et al. 's description of specific biological processes [22].More details were showed in Supplementary file 1.

### Multiomics data analyses

Differences in somatic mutations, CNVs and DNA methylation among three clusters were performed to comprehend the molecular characterization and differences among these three subtypes. Detailed analysis procedures are provided in Supplementary file 1.

### Dimension reduction and generation of the NII Score

For transformation from qualitative clustering to quantitative models, we developed an NII score system based on NII.cluster. Detailed analysis procedures are provided in Supplementary file 1. Finally, we applied the gene expression grade index to define the NII score of each patient:

$$\mathrm{NII\ score}=\sum \left(\mathrm{PCBi}-\mathrm{PCAi}\right)$$

### Construction of integrated prognostic models

By using the R package rpart and the NII score, a decision tree was constructed to stratify risks based on recursive partitioning analysis(RPA).Detailed analysis procedures are provided in Supplementary file 1.

### TME characteristics, chemotherapy and immunotherapy response prediction of NII score subtypes

To further characterize the correlation between the NII score and TME, methods for evaluating and quantifying the TME mentioned above were assessed. Then, functional enrichment analysis was executed to demonstrate signaling pathway heterogeneity. Detailed analysis procedures are provided in Supplementary file 1.

### Cell cultures and short hairpin RNA knockdown of VCAM1 in GC cells

The GC cell line SNU-216,HGC-27 and SNU-1 were purchased from American Type Culture Collection(ATCC), and NCC-24 was purchased from Korean Cell Line Bank. The GC cells were cultured in RPMI-1640 (Gibco,C11875500BT) with 10% fetal bovine serum, penicillin (100U/ml), and streptomycin (100 g/ml) in a humidated incubator with 5% CO_2_ at 37 ℃. Lentiviral shRNA and overexpression vectors targeting VCAM1 were purchased from GeneChem(Shanghai,China).The inhibitors of STAT3 phosphorylation(MCE,HY-13818) was added in culture medium to stimulate GC cells for one day.

### Cell proliferation and motility in GC cells

Cell proliferation were performed with Cell Counting Kit-8(Beyotime,C0041) in 37 ℃,5% CO_2_ for 1 h and tested by microplate reader(HBS-1101) at 450 nm. Motility analysis was performed in Transwells system(Corning,3422) with 5% CO_2_ at 37 ℃ for 48 h,and the fixation and staining were under manufacturer’s protocol.

### Western blot, immunohistochemical, immunofluorescence and qPCR analyses

Cancer cells were collected by treatment with trypsin(Gibco,25,200,072).Tumor tissues were processed prior to lysis using a tissue grinder(JXFSTPRP,CLN-24) at 4℃.The protein was extracted in RIPA buffer(EpiZyme,PC102) containing a complete protease inhibitor cocktail(EpiZyme,GRF101) and phosphatase inhibitors (EpiZyme,GRF102).Western blots were performed using the primary antibodies listed in Supplementary file 1.

Tissue sections and antigen retrieval were deparaffinized according to standard protocols.Preblocking was performed with goat serum(ZSGB-bio,ZLI-9022). The primary antibodies were used for immunohistochemistry and listed in Supplementary file 1. DAB staining(ZSGB-bio,ZLI-9018) and fast-red staining(ZSGB-bio,ZLI-9045) were used to stain markers in brown and red, respectively. Multiple fluorescent targets were enhanced with the tyramide signal amplification(TSA) staining system and listed in Supplementary file 1. Images were obtained with an LSM980(ZEISS) confocal microscope.

TRIzol reagent (15,506,026, Gibco) was used to extract total RNA from cancer cells.The cDNA was prepared using the High-fidelity cDNA Synthesis kit (Accurate Biology,AG11706) according to the manufacturer’s protocol.Quantitative RT‒PCR gene expression analyses were performed with QuantStudio 5(Applied Biosystems,A28139).The primers were listed in Supplementary file 1. Gene expression data were normalized to GAPDH mRNA expression and are presented as 2∆CT.

### Statistical analysis

The Kaplan‒Meier method and log-rank test were used to generate survival curves to judge differences between groups.The Wilcoxon test was used for comparisons of two groups.Kruskal–Wallis and one-way analysis of variance tests were used when comparing more than two groups.Clinical information was analyzed using chi-squared or Fisher's exact tests. R-4.0.5 (https://www.r-project.org/) was used for all statistical analyses.We conducted all comparisons two-sided with an alpha level of 0.05 and applied the Benjamini–Hochberg method to control the false discovery rate(FDR).

## Results

### Construction, verification and universality of the inflammation score

**Fig. 1:**
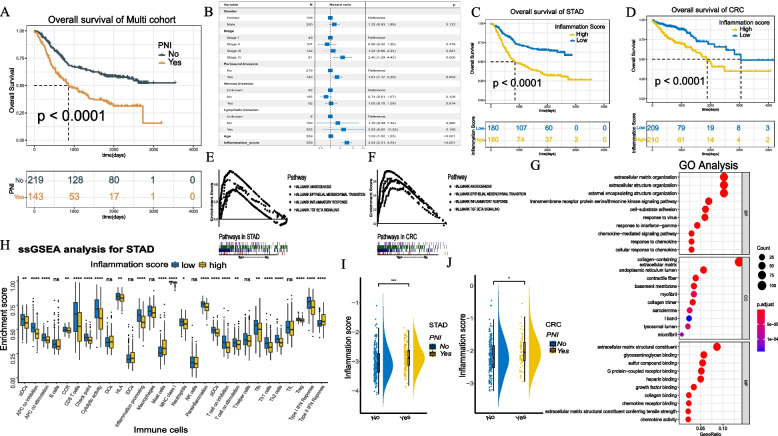
**A** Overall survival curves for of all GC patients in the training cohort. **B** Multivariate Cox regression analyses of significant prognostic factors. **C**, **D** Kaplan–Meier curves for the patients with high/low inflammation score in the training cohort and CRC cohort. **E**, **F** Gene Set Enrichment Analysis (GSEA) of high/low inflammation score groups in the training cohort and CRC cohort. **G** GO enrichment analysis of the significantly enriched biological processes between high and low inflammation score groups. **H** Derived ssGSEA scores of immune signatures obtained from STAD gene expression data for the groups of high and low inflammation score. **I**, **J **The comparing of Inflammation score between PNI and non-PNI groups in the training cohort and CRC cohort

First, we confirmed a significant difference in survival between patients with PNI and those without PNI in the training cohort and Nanfang cohort 1(*P* < 0.001, Fig. 1A, *P* = 0.02, Figure S1A).Patients with PNI had a worse prognosis. We constructed a model to measure the inflammatory level of patients based on the results of univariate analysis (Table S4) among 200 inflammatory response-related genes (Table S2) altered in GC patients with or without PNI. Afterward, we obtained 22 prognosis-related candidate genes (Table S3) from the three clusters, and provided an inflammation score for each patient with the LASSO Cox regression analysis model (Figure S1B-D).Patients were stratified into low or high inflammation score groups based on the medium value. As shown in Fig. 1B, age,PNI and the inflammation score were independent prognostic factors(*P* < 0.001).On the one hand, the prognostic difference was considerable in two groups in survival analysis (Fig. 1C).On the other hand,comparing the low and high inflammation score groups, biological cytology, angiogenesis, epithelial mesenchymal transition, inflammatory response and TGF- signaling were enriched in GSVA (Fig. 1E), and extracellular matrix organization, extracellular structure organization, external encapsulating structure organization and collagen containing extracellular matrix were enriched in GO analysis (Fig. 1G). For ssGSEA, significant differences in the immune microenvironment were observed between the low and high inflammation score groups. More CD8^+^ T lymphocytes and T helper cells were noted in the low inflammation score group (Fig. 1H).For the ESTIMATE-related score, the ImmuneScore of high-inflammation score group was significantly lower than that in the low group, while the StromalScore were in direct contradiction (Figure S1E). In addition,PNI patients had higher inflammation scores than patients without PNI (Fig. 1I). The proportion of two groups in patients with or without PNI is shown in Figure S1F. These analyses were also applied to patients with CRC, and the results were consistent (Fig. 1D, F, J).

### Transcriptome traits of NII cluster subtypes

#### Development and validation of the NII cluster system

**Fig. 2:**
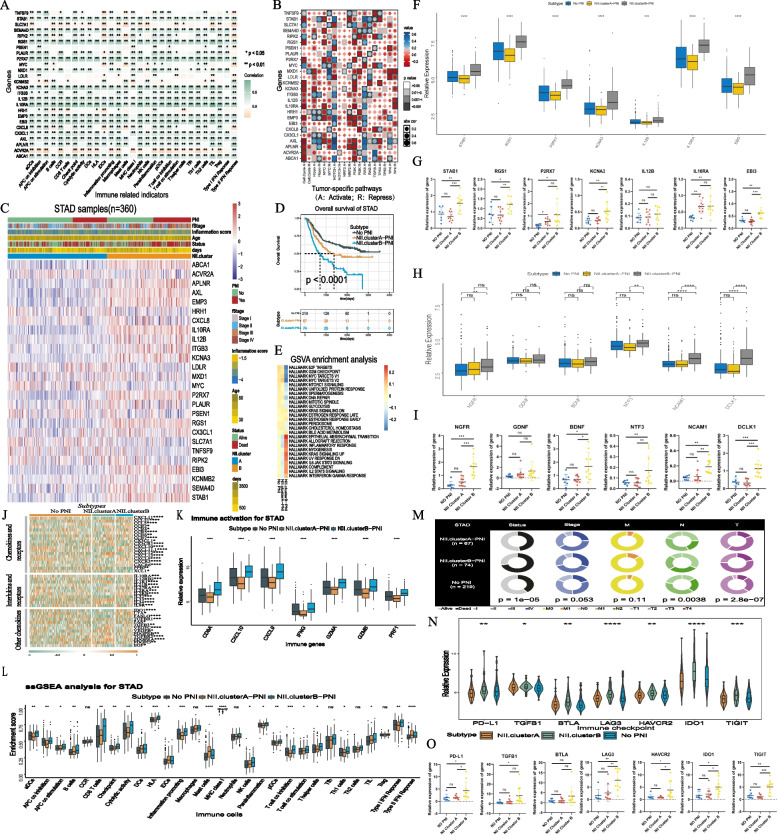
**A** The correlation between 26 inflammation-related genes and immune-related signatures. **B **The correlation between the expression level of the 26 inflammation-related genes and crucial tumor-specific pathways is shown in the heatmap. **C** Unsupervised clustering of 26 inflammation-related genes in the training cohort. The distribution of clinicopathological characteristics, including age, survival status, overrall survival, PNI, inflammation score and TNM stage, as well as the NII.cluster, are shown above. Rows represent genes, and columns represent samples. **D** Kaplan–Meier curves for overall survival (OS) of all GC patients in three subtypes(NII.clusterA-PNI, NII.clusterB-PNI and No PNI) (Log rank test, *p* < 0.0001). **E** GSVA analysis reveals enriched vital signal pathways in HALLMARK among three subtypes. Rows and coloumns are defined by the HALLMARK signal pathway and consensus scores for each subtype, respectively. **F**, **G** The genes expression of STAB1, RGS1, P2RX7, KCNA3, IL12B, IL10RA and EBI3 in different NII clusters of training cohort and Nanfang cohort2(RT-PCR).The asterisks represented the statistical P-value (*P < 0.05; **P < 0.01; ***P < 0.001). **H**, **I** The PNI related marker expression in different NII clusters of training cohort and Nanfang cohort2(RT-PCR).The asterisks represented the statistical P-value (*P < 0.05; **P < 0.01; ***P < 0.001). **J** The thermogram exhibits variations in gene expression of chemokines, interlukins and other cytokines among the three subtypes (Kruskal–Wallis test). Asterisk indicates P-value(*P < 0.05; **P < 0.01; ***P < 0.001). **K** The expression of immune-activation-relevant genes (CD8A, CXCL10, CXCL9, IFNG, GZMA, GZMB, PRF1) among three subtypes. (L)The fraction of tumor-infiltrating immune signatures calculated by ssGSEA algorithm in three subtypes. Within each subtype, the scattered dots represent immune-signature values. The asterisks represented the statistical P-value (*P < 0.05; **P < 0.01; ***P < 0.001). **M** Pie charts showing the Chi-squared test of clinicopathologic factors for three subtypes in the Multi-cohort. **N**, **O** The comparing of immune-checkpoint genes in three subtypes of training cohort and Nanfang cohort2(RT-PCR), including PD-L1, TGFB1, BTLA,LAG3, HAVCR2, IDO1, TIGIT. The asterisks represented the statistical P-value (*P < 0.05; **P < 0.01; ***P < 0.001)

In the training cohort, batch univariate Cox regression analysis for patients with and without PNI was performed to identify inflammation-related genes with significant prognostic values,including 48 genes in PNI patients, and 66 genes in non-PNI patients (Table S4). Twenty-six candidate PNI-related specific inflammatory genes were selected from the Venn diagram (Figure S2A). We investigated the biological significance of these genes in analysis of infiltrating immunocytes and pathways. Noticeably, we found STAB1, RGS1, P2RX7, KCNA3, IL12B, IL10RA and EBI3 had strong correlation with infiltrating immunocytes,which were regarded as factors in extensive immune activation (Fig. 2A). In Fig. 2B, the activity of tumor-specific pathways, such as the cell cycle, NOTCH and RAS pathways, was closely related with the expression of selected genes.

Based on 26 genes, two NII clusters(NII.clusterA and NII.clusterB) were divided with unsupervised clustering in the training cohort and Nanfang cohort 2 (Figs. 2C and S2C). The clustering process at different k values were shown in Figure S2B. The weight of 26 genes in clustering was shown in Figure S2D. Based on the survival analysis of patients with and without PNI (Figure S2E and S2F), three subtypes were divided: NII.clusterA-PNI, NII.clusterB-PNI and No PNI. Subgroup analysis of the above mentioned immune activation genes(STAB1, RGS1, P2RX7, KCNA3, IL12B, IL10RA and EBI3) in training cohort and Nanfang cohort2 showed that these genes were significantly up-regulated in NII.clusterB-PNI (Fig. 2F and G).We further examined PNI-related markers in these three subgroups with reference to other reported PNI-related markers [23–26](BDNF,GDNF,NGFR,NTF3,NCAM1 and DCLK1),and found most PNI-related signatures were also up-regulated in NII.clusterB-PNI (Fig. 2H and I).Marked survival differences were observed among subtypes(Fig. 2D). Then, we analyzed the differentially expressed genes of the two subtypes of PNI, obtaining 357 downregulated genes and 1200 upregulated genes (Figure S2G and Table S5, FC = 1.4 and adj.*P* < 0.05).

#### The biological and TME characteristics of NII subtypes

We compared NII subtypes in PNI by GO, KEGG (Table. S6) and GSVA analysis (Fig. 2E). The same analysis of comparison with the No PNI group is shown in Table S5, Table S6 and Fig. 2E. Summarizing the results above, most inflammatory signaling pathways were significantly enhanced in NII.clusterB-PNI, such as epithelial mesenchymal transition (EMT), KRAS,and inflammatory response.

In the analysis of immune-related factors, the expression of chemokines, interleukins and other cytokines was significantly different among the three subtypes (Fig. 2J).Further,we compared the differences in infiltrating immunocytes and immunity activation factors among the three subtypes (Fig. 2K, L).The results hinted at great variation in the immune microenvironment.The results of ESTIMATE-related indicators were consistent: scores were the highest in NII.clusterB followed by non-PNI group,and the lowest scores were noted in NII.clusterA (Figure S2H). In addition,we analyzed the expression of immune checkpoints among the three subtypes in training cohort and Nanfang cohort2 (Fig. 2N, O).The relationship between subtypes and the TNM system is shown in Fig. 2M.The division of subtypes was significantly related to lymphatic metastasis, infiltration degree and survival(all *P* < 0.001).

#### Noteworthy differentially-expressed genes and pathways among NII cluster subtypes

**Fig. 3:**
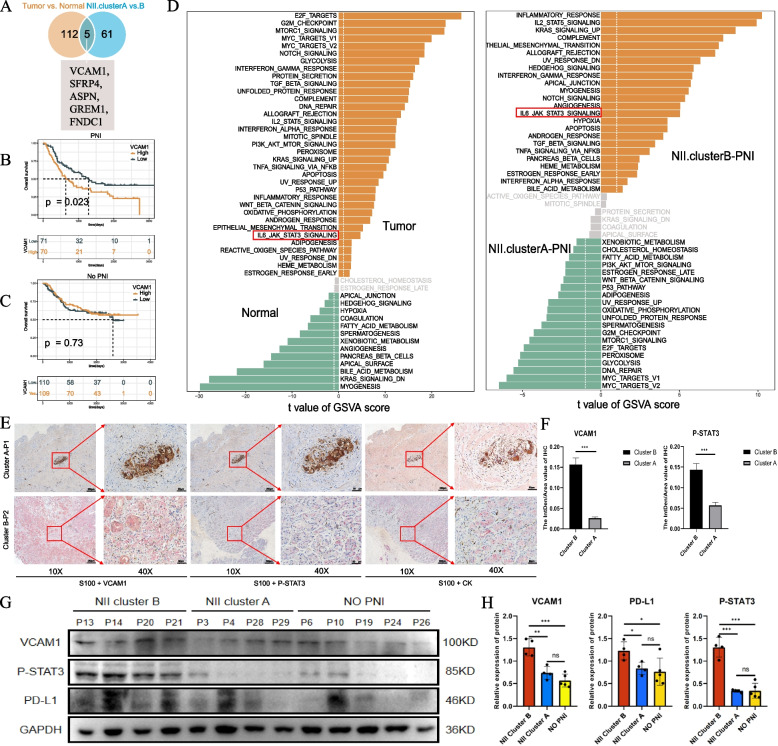
**A** Five overlapping genes (VCAM1, SFRP4, ASPN, GREM1 and FNDC1) in the intersection of “Tumor vs. Normal” and “NII.clusterA-PNI vs. NII.clusterB-PNI” are considered as genes playing potential regulatory roles in the inflammation mediated by perineural invasion. **B**-**C** Kaplan–Meier curves for the patients with high and low VCAM1 expression in PNI group (Log rank test, *p* = 0.023.) and No PNI group(Log rank test, *p* = 0.73.) **D** A marked signal pathway (IL6-JAK-STAT3 SIGNALING) tabbed by red box is regarded important in the inflammation mediated by perineural invasion. **E** Representative IHC results of VCAM1, P-STAT3 and CK in tumor slices of NII.clusterA-PNI and NII.clusterB-PNI patients.(S100 marked nerves in brown,VCAM1,P-STAT3 and CK were in pink) **F** The statistical results of VCAM1 and p-STAT3 in (E).(All *P* < 0.001) **G**, **H** The WB results of VCAM1,P-STAT3 and PD-L1 protein expression of tumor tissue from No PNI,NII.clusterA-PNI and NII.clusterB-PNI patients.Statistics are based on the average of the gray values of the bands from three independent experiments.The asterisks represented the statistical P-value (*P < 0.05; **P < 0.01; ***P < 0.001)

To determine the specific molecular characteristics of neuroinflammation with PNI, we obtained 5 gene signatures from a Venn diagram between the DEGs of Tumor vs. Normal (Figure S3A) and NII.clusterA-PNI vs. NII.clusterB-PNI (Table S5), including VCAM1, SFRP4, ASPN, GREM1 and FNDC1 (Fig. 3A).However, only the VCAM1 survival analysis was statistically significant in PNI (Fig. 3B, C and Figure S3E-S3H).VCAM1 expression was also significantly different in other validation sets(Figure S3B-S3D).Further,we noticed that IL6-JAK-STAT3 SIGNALING was enriched in both tumor and NII.clusterB-PNI (Fig. 3D).This pathway is reported to be closely related to VCAM1 in past reports [27].

**Fig. 4:**
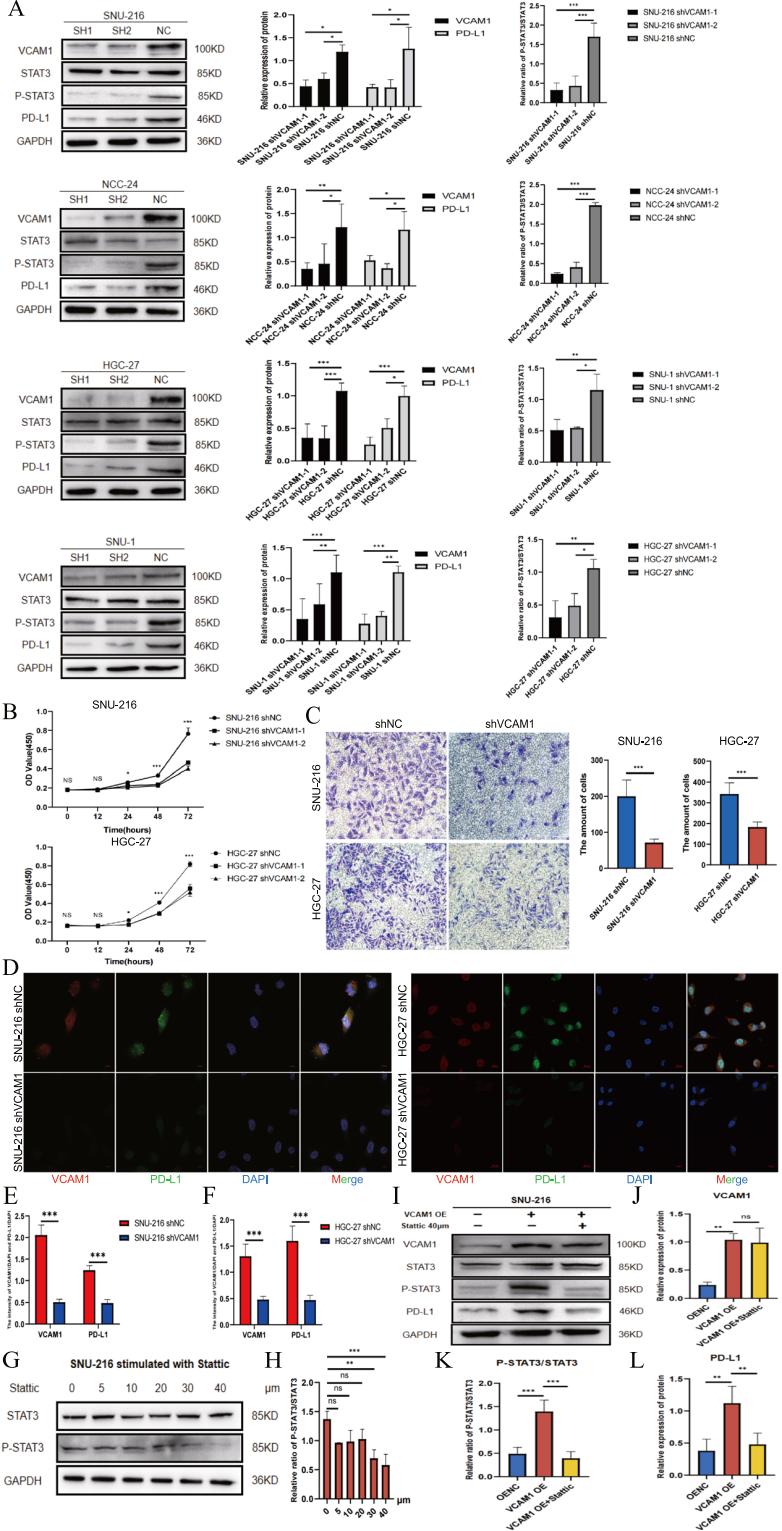
**A** VCAM1,STAT3, P-STAT3 and PD-L1 protein expression of shRNA cell models in SNU-216,NCC-24,HGC-27 and SNU-1(VCAM1 sh1,sh2 and natural contrast).Statistics are based on the average of the gray values of the bands from three independent experiments.The asterisks represented the statistical *P*-value (*P < 0.05; **P < 0.01; ***P < 0.001). **B** The OD value of CCK8 analysis of VCAM1 silencing of three independent experiments in SNU-216 and HGC-27(shVCAM1 vs. shNC,*P < 0.05; **P < 0.01; ***P < 0.001) **C** The representative figures of transwell experiments of SNU-216 and HGC-27 for culturing 48 h.The statistical results were performed in 5 random views of per group under 20X. (*P < 0.05; **P < 0.01; ***P < 0.001) (**D**) The representative immunofluorescence figures of SNU-216 and HGC-27(shVCAM1 vs. shNC,VCAM1 in red,PD-L1 in green and DAPI in blue,under 40X). **E**, **F** The statistical results of VCAM1 and PD-L1 in (**D**). (*P < 0.05; **P < 0.01; ***P < 0.001). **G** The WB results of SNU-216 stimulated with stattic. **H** The statistical result of three independent experiments in (**G**). (*P < 0.05; **P < 0.01; ***P < 0.001). **I** The WB results of SNU-216 VCAM1-OE stimulated with stattic. **J**, **K**, **L** The statistical result of three independent experiments in (**I**). (*P < 0.05; **P < 0.01; ***P < 0.001)

Immunohistochemistry(IHC) analysis of VCAM1, P-STAT3 and cytokeratin(CK) was performed in NII.clusterA-PNI and NII.clusterB-PNI samples (Fig. 3E). VCAM1 and P-STAT3 were also highly expressed in NII.clusterB-PNI compared with NII.clusterA-PNI (Fig. 3F). In the analysis of Western Blot(WB) of tumor tissue,VCAM1,p-STAT3 and PD-L1 in NII.clusterB-PNI were up-regulate than that in other subtypes (Fig. 3G, H).In addition,we successfully constructed VCAM1 shRNA cell models of the GC cell lines SNU-216,NCC-24,HGC-27,SNU-1 (Supplementary Figure S10A-D). When VCAM1 expression was downregulated, STAT3 phosphorylation was inhibited,and PD-L1 was also be down-regulated (Fig. 4A).

Further,CCK8 cell proliferation assay and transwell cell shuttle assay were performed on shVCAM1 and shNC gastric cancer cells.VCAM1 silencing will suppress the cell proliferation and motility of cancer cell.(All *P* < 0.001, Fig. 4B, C) In the results of immunofluorescence between shVCAM1 and shNC,we could also observe that PD-L1 was down-regulated in shVCAM1 GC cells compared with shNC GC cells.(All *P* < 0.001, Fig. 4D, E, F) To validate the factor of VCAM1 in regulating the PD-L1 expression by affecting STAT3 phosphorylation, we successfully constructed VCAM1 overexpression model in GC cells (Supplementary Figure S10E) and used stattic to inhibit STAT3 phosphorylation in gastric cancer cells.In Fig. 4G-H,STAT3 phosphorylation could be inhibited well at 40 μm of stattic in SNU-216.In Fig. 4I-L,we could observe p-STAT3 and PD-L1 were up-regulated in VCAM1-OE,while that process were stopped by stattic.

#### Mutational and methylation features of NII clusters

**Fig. 5:**
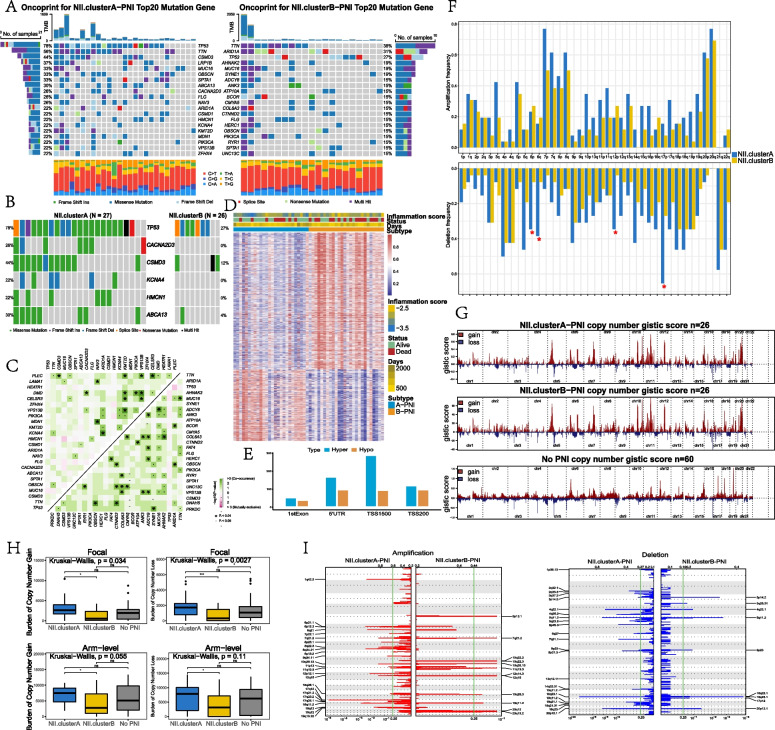
**A** Mutation landscape of NII.clusterA-PNI and NII.clusterB-PNI subtypes. The 20 genes with the highest mutation frequency are shown and samples are sorted by the TMB in each subtype. The small figure above shows the TMB, the numbers on the right exhibit the mutation frequency of each regulator, and the figure laterally shows the proportion of each variant. **B** Waterfall plot reveals significantly differentially mutated genes between NII.clusterA-PNI and NII.clusterB-PNI subtypes(Fisher exact test, p < 0.05). Individual patient is represented in each column. The numbers on either hand show the mutation frequency of each gene. Different colors represent different mutation modes. **C** Interaction effect of genes mutating differentially in patients in the NII.clusterA-PNI and NII.clusterB-PNI subtypes. **D** Heatmap of differentially methylated CpG sites in the promoter region between samples of NII.clusterA-PNI and NII.clusterB-PNI subtypes. **F** The diversity of methylation of the different regions of genes in the promoter region including 1stExon, SUTR, TSS1500 and TSS200. **G** Comparisons of arm-level amplification and deletion frequencies between NII.clusterA-PNI and NII.clusterB-PNI subtypes. **H** Copy number profiles for three subtypes, with gains in orange and losses in green. Gene segments are placed according to their location on chromosomes, ranging from chromosome 1 to chromosome 22. **I** Distribution of CNV with focal-level and arm-level copy number alterations among three subtypes. (ns *P* > 0.05, *P < 0.05, **P < 0.01, ***P < 0.001, ****P < 0.0001) Detailed cytoband with focal amplification (up) and focal deletion (down) in NII.clusterA-PNI and NII.clusterB-PNI

For somatic mutations, specifically mutated genes as well as mutation types in the top 20 genes of the 3 subtypes were shown in Fig. 5A and Figure S4A. A higher mutation frequency was noted in NII.clusterA-PNI compared with NII.clusterB-PNI. For instance,in NII.clusterA-PNI, TP53 showed the highest mutation frequency(78%) followed by TTN(56%) and CSMD3(44%), and missense mutations were the most common. In contrast, TTN(38%), ARID1A(31%), and TP53(27%) were the top three most frequent mutations in NII.clusterB-PNI. Genes with significantly differential mutations between subtypes NII.clusterA-PNI vs. NII.clusterB-PNI were selected for comparison in Fig. 5B. The same analysis of No PNI vs. NII.clusterA-PNI and No PNI vs. NII.clusterB-PNI are shown in Figure S4B. In the analysis of the cooccurring and exclusive mutation patterns,distinct frequent comutations were noted in NII.clusterB-PNI (e.g.,MUC16-ADCY8, AHNAK2-COL6A3, and CTNND2-DNAH5) and NII.clusterA-PNI (e.g., MDN1-NAV3, DMD-VPS13B, and CELSR3-PLEC) (Fig. 5C). The same analysis was performed for the non-PNI group (Figure S4C).Arm-level deletion frequencies in 6p, 6q, 12p and 17p were not noteworthy distinctions between NII.clusterA-PNI and NII.clusterB-PNI (Fig. 5F). The results of non-PNI vs. NII.clusterA-PNI and non-PNI vs. NII.clusterB-PNI are shown in Figure S4H-I. According to Fig. 5G, the distribution of G-scores on chromosomes 1–22 of subtypes was shown.The burden of copy number gain and loss between NII.clusterA-PNI and NII.clusterB-PNI at both focal and broad levels showed a remarkable difference, whereas the non-PNI subgroup showed no apparent discrepancy(Fig. 5H). Detailed cytobands with focal amplification(upside) and focal deletion(downside) of NII.clusterA-PNI and NII.clusterB-PNI were shown in Fig. 5I. The results of the non-PNI group are shown in Figure S4F-S4G.

Regarding methylation, the heatmap showed the distribution of differential CpG sites in NII.clusterA-PNI and NII.clusterB-PNI (Fig. 5D).The results indicated that patients in NII.clusterB-PNI have more hypermethylated sites. In addition, the proportions of hypermethylated and hypomethylated sites in the screened promoter regions(5' UTR, TSS200, TSS1500 and 1st Exon) are shown in Fig. 5E. NII.clusterB-PNI patients had more highly differentially methylated sites in four promoter regions compared with NII.clusterA-PNI patients.Similarly, we present a comparison of the non-PNI and NII.clusterB-PNI subtypes in Figure S4D-E. Patients in NII.clusterB-PNI have more differentially hypermethylated sites.

#### The analysis of inflammation score and NII cluster subtypes in race,TCGA classification and ACRG classification

**Fig. 6:**
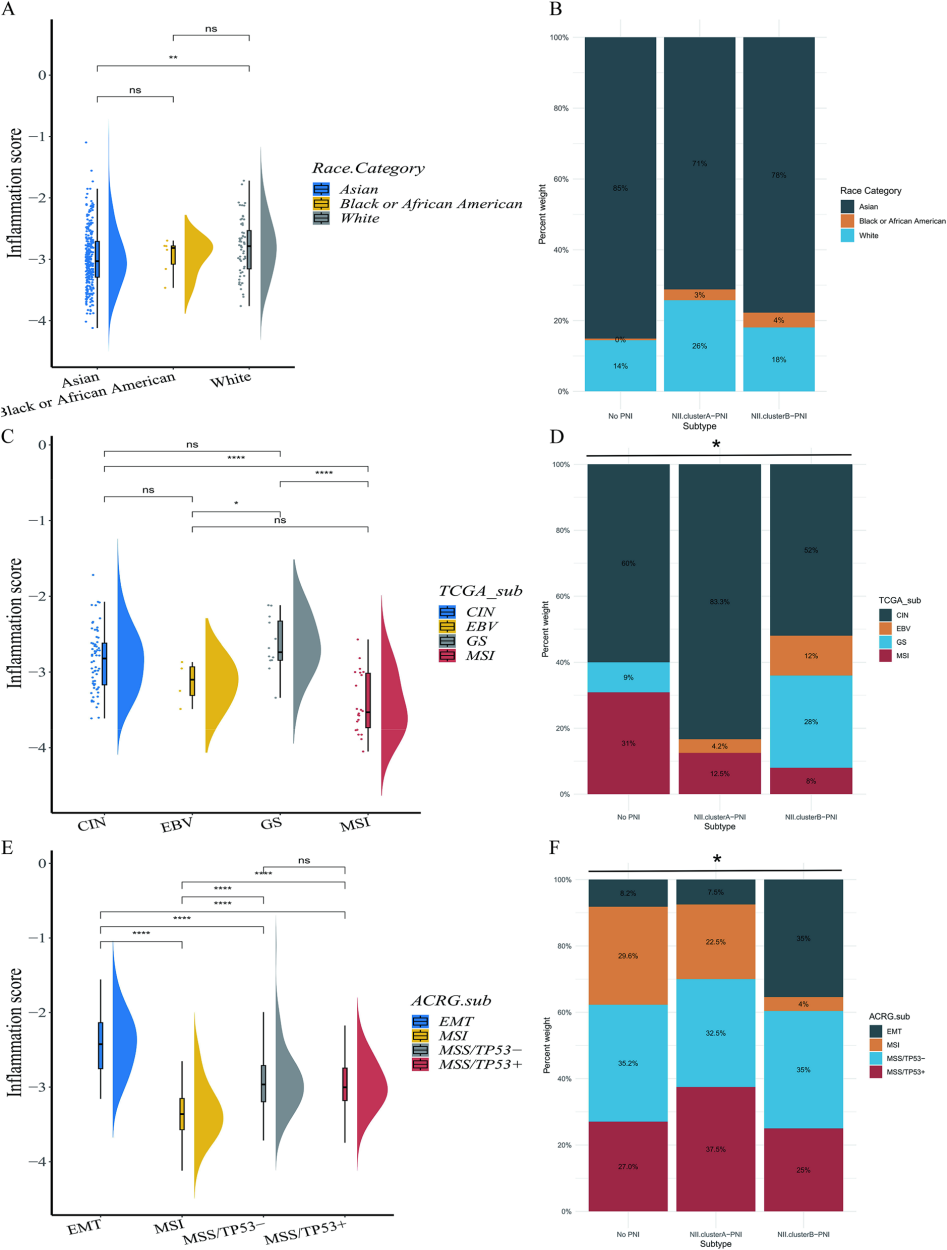
**A** The analysis of inflammation score among different races in training cohort. **B** The distribution of different races in NII classification in training cohort. **C** The analysis of inflammation score among TCGA subtypes in training cohort. **D** The distribution of TCGA subtypes in NII classification in training cohort. **E** The analysis of inflammation score among ACRG subtypes in training cohort. **F** The distribution of TCGA subtypes in NII classification in training cohort.(All of above,ns *P* > 0.05, *P < 0.05, **P < 0.01, ***P < 0.001, ****P < 0.0001)

In order to learn the relation of inflammation and dietary or living habits,we performed inflammation score among the white,the Asians and the Black or African American.In GC patients, the white people have a higher inflammatory burden in the tumor microenvironment than the Asians, while the Black or African American people have an intermediate inflammatory burden level between the two groups (Fig. 6A).In No PNI, NII.clusterA-PNI, and NII.clusterB-PNI, the distribution of these three groups of people is also different (Fig. 6B, *P* < 0.05).

Further,we have performed inflammation score and NII cluster subtypes analysis in TCGA classification and ACRG classification.In TCGA classification,genomically stable (GS) had the highest inflammatory burden (Fig. 6C), while microsatellite instability (MSI) had the lowest. In the distribution results of TCGA classification in NII cluster subtypes (Fig. 6D), GS was mainly distributed in NII.clusterB-PNI.MSI was mainly distributed in No PNI and NII.clusterA-PNI.As for ACRG classification (Fig. 6E),EMT had the highest inflammatory burden,while MSI had the lowest.The majority of EMT patients were distributed in NII.clusterB-PNI, in contrast, the majority of MSI patients were distributed in No PNI and NII.clusterA-PNI (Fig. 6F).

#### Construction and validation of the NII scoring system

**Fig. 7:**
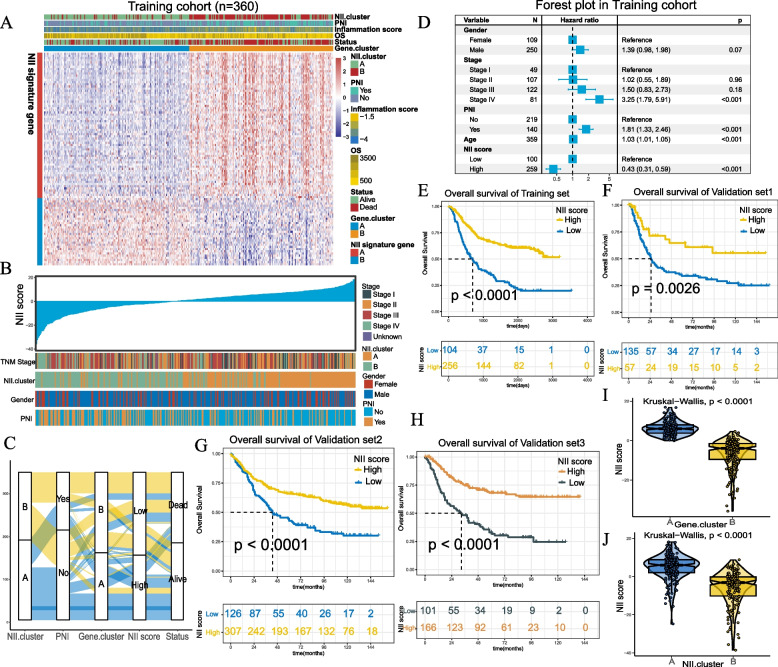
**A** Identification of NII score subgroups of STAD patients. **B** An overview of the association between known clinical and inflammation features (TNM stages, NII.clusters, gender and PNI) and NII score. Columns represent samples sorted by NII score from low to high (top row). Rows represent known clinical and inflammation features. **C** Alluvial diagram of NII.clusters in groups with different PNI groups, Gene.clusters, NII score, and survival status. **D** Forest plot displays the result of multivariate Cox regression analyses of significant prognostic factors. (Log rank test *p* < 0.001.) **E**–**H** Kaplan–Meier analyses demonstrate that patients with higher NII score exhibit worse prognosis in the training cohort (*P* < 0.0001), validation cohort 1 (*P* = 0.0026), validation cohort 2 (*P* < 0.0001) and validation cohort 3(*P* < 0.0001). **I**, **J** Relative distribution of NII score in groups with Gene.clusters and NII clusters. The thick line represents the median value. The bottom and top of the boxes are the 25th and 75th percentiles (interquartile range). The differences between groups were both compared through the Kruskal–Wallis test (*p* < 0.0001)

To predict the prognosis of and immune infiltration level in STAD patients,we generated the NII score as a quantitative indicator of the NII landscape using principal component analysis(PCA).In the training cohort,we obtained 1207 upregulated genes and 351 downregulated genes by comparing NII.clusterA and B (Figure S5A and Table S5). A detailed description of the enriched biological pathways is provided in Table S6. Then 47 representative genes were ultimately selected for PCA construction by univariate Cox regression (*p* < 0.05) and random forest(ntree500, nPerm50) (Table. S7, Figure S5B). Unsupervised clustering was performed in the classification of STAD patients(Gene clusters A and B) and representative genes(NII gene signatures A and B)(Fig. 7A). The prognosis between gene cluster A and B was remarkably different in training cohort (Figure S5C). Finally,each patient acquired an individual NII score according to PCA. We ranked the GC samples according to their NII score and analysed correlativity with other factors (Fig. 7B). The score construction process and its relationship with PNI, survival status and other clustering methods were shown in Fig. 7C. Based on the training cohort, the best cutoff value was -3.105 which was used to divide high or low NII score.The NII score manifested as a remarkable prognostic indicator via multivariate regression analysis in the training cohort as well as the other three validation cohorts (Fig. 7D, S6A-S6C). Subgroup analysis verified the independence of the NII score (Figure S6D-S6G). There were considerable differences in survival analysis between high and low NII score subgroups (Fig. 7E-H). Furthermore, the prognostic power of the NII score was examined in a wide spectrum of gastrointestinal tumors (Figure S5D-S5I). In addition, we discovered that the NII scores of NII.clusterA and Gene cluster A were both increased (Fig. 7I, J).

#### Analyse of immunotherapy and chemotherapy sensitivity for NII score system

**Fig. 8:**
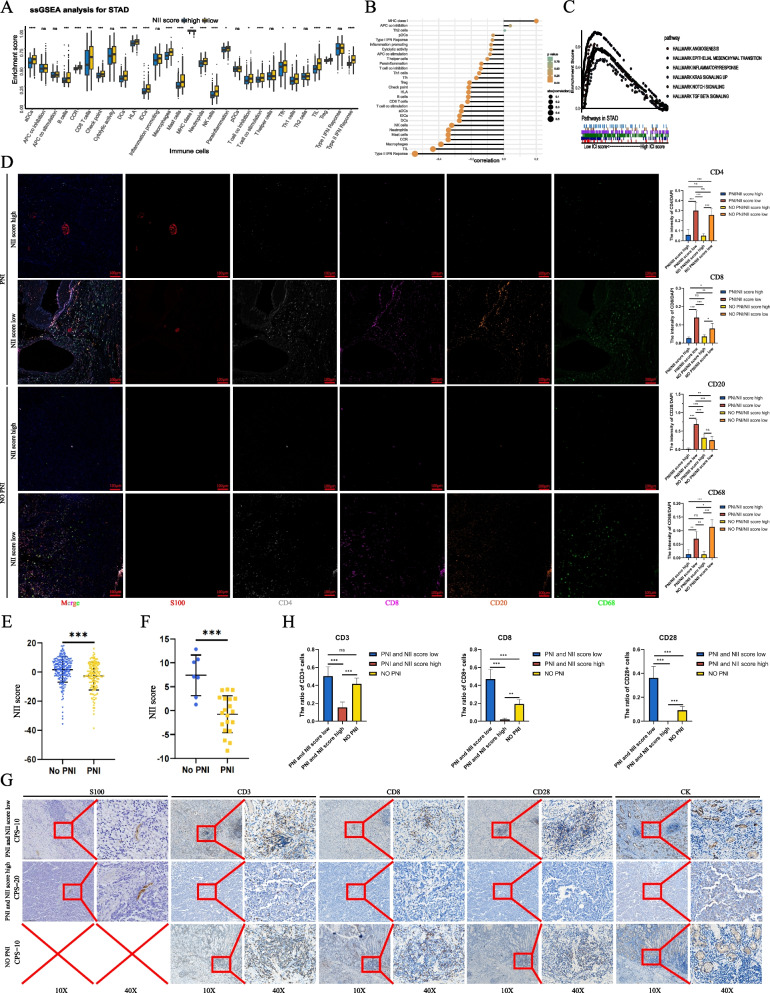
**A** Derived ssGSEA scores of immune signatures obtained from STAD gene expression data for the groups of high and low NII score. The range of P values were labeled above each boxplot with asterisks.(ns *P* > 0.05, *P < 0.05, **P < 0.01, ***P < 0.001, ****P < 0.0001) **B** The correlation of immune cells and NII score in the training cohort. The range of P values are represented by color from yellow to green. **C** The significantly enriched signal pathways from Gene Set Enrichment Analysis (GSEA) performed between the subgroups of high and low NII score in the Multi cohort. **D** The representative results of multiple immunofluorescence staining of subgroups(PNI with high NII score,PNI with low NII score,non-PNI with high NII score and non-PNI with low NII score).(S100 in red,CD68 in green,CD20 in orange,CD8 in purple,CD4 in white and DAPI in blue.The statistical results were performed in 5 random views of per group under 40X. *P < 0.05; **P < 0.01; ***P < 0.001) **E**, **F** The comparing between PNI and non-PNI patients in training cohort and Nanfang cohort 2. **G** The representative figures of IHC analyse of subgroups(PNI with high NII score, PNI with low NII score and non-PNI patients, CPS scores were obtained from clinical pathological report) accepting anti-PD1 treatment. (S100,CD3,CD8,CD28 and CK were stained with DAB in brown,nucleus were stained with hematoxylin in purple) (H)The statistical result of CD3,CD8 and CD28 were performed in 5 random views of per group under 40X.(*P < 0.05; **P < 0.01; ***P < 0.001)

In Fig. 8A and B, though more abundant immunocytes infiltrated in low NII score patients, these cells also exhibited more immunosuppressive characteristics, including multiple immune checkpoints and Treg cells. Similarly, patients with low NII scores had higher immune scores, stromal scores and ESTIMATE scores (Figure S7A). We found that the angiogenesis signaling pathway, epithelial mesenchymal transition signaling pathway, inflammatory-response signaling pathway, TGF-β signaling pathway, Notch signaling pathway and other immune response pathways were activated in the low NII score subgroup (Fig. 8C). To analyze the relationship between the NII score and PNI, we found patients without PNI distinctly obtained higher NII scores (Fig. 8E, F), and their prognosis was subsequently more optimistic. The scores of each patient in the training cohort and Nanfang cohort 2 were listed in Tables S9, S10. The non-PNI group exhibited a higher frequency(72% vs.52%) compared with the high NII score subgroup (Figure S8A). In the representative results of multiple immunofluorescence staining (Fig. 8D), patients with a low NII score had more immune cell infiltration than those with a high NII score (DAPI, Figure S8B-S8E). Similar findings are noted in Fig. 8A. In addition,we analyzed the immunotherapy response in the tissue of GC patients after anti-PD1 treatment (Fig. 8G, CPS score obtained from pathological report), and found CD3 + /CD8 + /CD28 + T cell infiltration increased in the TME of PNI patients with a low NII score and the non-PNI patients (Fig. 8H). Upon anti-PD1 treatment, no CD8 + /CD28 + T cells were noted in PNI patients with a high NII score,and tumor cells(CK marked, Fig. 8G) were still diffused in the TME. Various degrees of T-cell activation were noted in other groups, and tumor cells could be seen in small clusters.

Based on the CTRP database and PRISM database, we report some potentially effective chemotherapy drugs for high and low NII score patients (Figure S9A-S9C). The lower AUC and IC50 values of high NII score patients indicate sensitivity to these drugs, suggesting that patients with high NII scores might benefit more from chemotherapy(including 5-fluorouracil,gemcitabine,oxaliplatin). More chemicals were shown in Figure S7B-S7C.

#### Generation and validation of integrated prognostic model

**Fig. 9:**
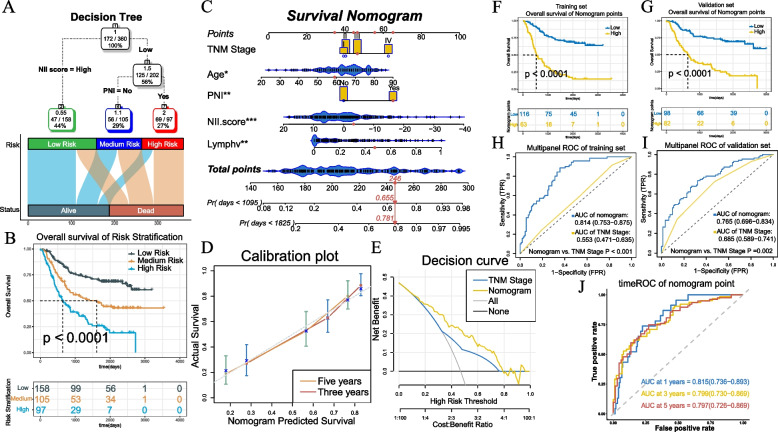
**A** A survival decision tree built to optimize the prognostic stratification combined with a alluvial diagram of risk stratification and survival status. **B** Significant differences of overall survival (OS) are observed among the three risk subgroups (*P* < 0.0001). **C** A personalized scoring nomogram is generated to predict 3- and 5-year OS probability with five parameters( TNM Stage, Age, PNI, Lymphv and NII score), and the arrow shows an example. **D** Calibration curves of 3-year and 5-year overall survival (OS) prediction are close to the ideal performance (45-degree line). **E** Decision curve demonstrates that the nomogram exhibited more powerful capacity of survival prediction compared with TNM stage system. **F**-**G** Kaplan–Meier curves for the patients with high and low overall survival of Nomogram points in the training cohort (Log rank test, *p* < 0.0001.) and validation cohort (Log rank test, *p* < 0.0001.). **H**-**I** The comparing between nomogram and TNM system with ROC in training cohort and validation cohort. **J** Time-dependent ROC curves of nomogram in training cohort

With the goal of optimizing prognostic stratification, we used the decision tree to establish three risk levels (Fig. 9A). Patients with high NII scores were defined as the low-Risk, whereas the medium- and high-risk levels were defined based on a low NII score & non-PNI and a low NII score & PNI, respectively. Significant differences in overall survival were observed among the three risk subgroups(*P* < 0.0001,Fig. 9B). In multivariate Cox analysis,the NII score,age,TNM stage,PNI and lymph node positive detection rate were independent factors significantly associated with OS (Table S8). Then, the personalized scoring nomogram was generated to predict 3- and 5-year OS probability (Fig. 9C). In the calibration curves, the 3- and 5-year survival predicted by the nomogram were consistent with the ideal performance (Fig. 9D). Decision curves indicated that the net benefits to patients offered by the nomogram surpassed TNM system (Fig. 9E). The nomogram appeared to be better at prominently predicting survival than TNM system in time-independent ROC analysis(training cohort: AUC of nomogram = 0.814 (0.753–0.875), AUC of TNM stage = 0.553(0.471–0.635),*P* < 0.001;validation cohort: AUC of nomogram = 0.765(0.696–0.834), AUC of TNM stage = 0.685(0.589–0.741), *P* = 0.002) (Fig. 9H, I). In the K-M curves(training cohort: *P* < 0.001; validation cohort: *P* < 0.001, Fig. 9F, G) and the time-dependent ROC curves (Fig. 9J), the formidable prognostic capacity of the nomogram was distinctly verified, and patients with higher nomogram points tended to worse prognosis.

## Discussion

PNI is a common pathological feature of solid tumors, which often leads to poor prognosis of patients [28–30]. PNI is listed as one of the recommended risk factors for chemotherapy in cancer guidelines [6, 7], even in the early stage of disease. Recently, the regulatory role of nerves in the tumor microenvironment has attracted the attention of researchers, especially their role in the regulation of tumor immunity. As an increasing number of immunotherapies have made breakthroughs in clinical trials, immunotherapy has gradually entered the clinic as a powerful means to treat cancer patients, such as the anti-PD1 regimen in GC [31–33]. However, a new challenge is currently noted. Immunotherapy is not effective in all patients enrolled in cohort studies or in clinical practice [34, 35]. The identification of patients who can benefit from treatment is urgently needed. In this study, we focused on GC patients with PNI and analyzed the relationship among PNI, inflammatory reactions and immunity. Based on these results, we developed a PNI-related scoring system and validated its performance in predicting the benefit of immunotherapy and chemotherapy in GC patients.

In this study, GC patients with PNI had significantly worse outcomes than patients without PNI, both in the public database cohorts and the Nanfang cohort. Our results are similar to those of most PNI-related cohort studies [28–30]. Inspired by the inflammatory response triggered by peripheral nerve damage, we assessed whether similar inflammatory infiltrations might occur in the PNI of the tumor microenvironment. Based on the inflammation score results, we found that patients with high levels of inflammation had a worse prognosis than those with low levels. The degree of the inflammatory response in GC patients with PNI was significantly higher than that in PNI-negative patients. In the GSEA results, we observed that inflammatory response pathways were enriched in PNI patients. Similarly, we obtained similar results from the TCGA cohort of colorectal cancer patients. In past studies, researchers have also suggested that perineural invasion is closely related to inflammatory infiltration in various cancers, such as in keratinocyte carcinomas [36] and pancreatic cancer [37]. Different degrees of inflammatory response induction potentially cause differences in immune cell infiltration in the tumor microenvironment.

As shown in Figure S1F, not all patients with PNI have a high inflammatory response, and not all negative patients have a low inflammatory response. It is possible that the type of inflammatory response caused by PNI has some unique characteristics.This notion seems to be supported by previous studies of cancer with PNI [38–40].Therefore, we further explored the relationship between PNI and the inflammatory response through unsupervised clustering.We found two PNI subtypes that exhibit opposite prognoses and significant differences compared with PNI-negative patients.In subsequent analysis, many pathways associated with malignant progression were highly enriched in NII.clusterB-PNI, such as EPITHELIAL MESENCHYMAL TRANSITION and KRAS SIGNALING. This finding also suggested that NII.clusterB-PNI patients had the worst prognosis, which was consistent with the survival analysis results. However, NII.clusterB-PNI also had the most abundant immune cell infiltration, including CD8 + T cells, B cells, DCs and APCs (Fig. 2L). In previous studies, abundant immune infiltration often predicts a good antitumor immune response [41, 42],which suggests a better prognosis. Interestingly, we found that most immune checkpoint markers,such as PD-L1, LAG3 and IDO1, were highly expressed in NII.clusterB-PNI (Fig. 2N, O). We also found that VCAM1 may represent a key molecular signature causing these differences among the three subtypes (Fig. 3). It has been reported that VCAM1 is closely related to PNI [43]. In the past, many reports have shown that VCAM1 and STAT3 are closely related, but the specific mutual regulation mechanism is not clear at present,especially in tumor microenvironment. Luo et al. [27] reported that IL6/STAT3 would promote VCAM1 expression in RAW264.7 in the cardiovascular system.In our results,we found that the VCAM1 expression of GC cells will also affect the phosphorylation of STAT3 in regulating PD-L1 expression.There seems to be a positive feedback regulation between VCAM1 and STAT3 in tumor cells. In addition,VCAM1 silencing will suppress the cell proliferation and motility of cancer cell,which were similar with Ye.et al. [44] reported in their study.These results explain the poor prognosis of NII.clusterB-PNI patients among the three subtypes.

In addition, we analyzed somatic mutations and DNA methylation among these subtypes.For somatic mutations, compared with NII.clusterB-PNI, NII.clusterA-PNI and No PNI patients had higher levels of somatic mutation frequencies.Comparisons revealed higher levels of arm-level amplification and deletion frequencies in NII.clusterA-PNI and No PNI patients compared with NII.clusterB-PNI patients.In previous reports, a copy number loss was related to the response to immune checkpoint blockade therapy [45].Cancer-specific neoepitopes may be generated by somatic mutations and deletion frequencies, and these may serve as good targets for cancer vaccines.More mutations might offer more opportunities for immunity against cancer [46], thus underscoring the better prognosis of NII.clusterA-PNI and No PNI patients than NII.clusterB-PNI patients. Regarding DNA methylation, it is worth noting that the promoter regions of BACH2 [47], MTAP [48], and RUNX1T1 [49] were hypermethylated in NII.clusterB-PNI patients compared with NII.clusterA-PNI patients.These genes act as inhibitory factors in cancer.

Considering the relationship between gastrointestinal tumors and the dietary or living habits of patients,we analyzed the inflammatory burden of gastric cancer patients of different races and their distribution in the NII cluster system (Fig. 6A, B). In GC patients, white people have a higher inflammatory burden in the tumor microenvironment than Asians, while Black or African American people have an intermediate inflammatory burden level between the two groups.In No PNI, NII.clusterA-PNI, and NII.clusterB-PNI, the distribution of these three groups of people is also different. These results suggest that different living or dietary habits may be related to the inflammatory burden in the tumor microenvironment of patients with GC.The H. Pilory-infection is also an important factor in causing chronic inflammation in GC [50].Unfortunately, we were unable to obtain a suitable cohort of H. Pilory-infected gastric cancer patients in public database with clear information of PNI. Therefore, this analysis could not be performed. Since the TCGA classification [51] and ACRG classification [52] of GC have been reported, these two classification methods have received extensive attention.We compared the differences in inflammatory burden across subtypes and their distribution in the NII cluster system.As reported in TCGA classification [51],PD-L1 was one of the molecular characteristics of EBV,we also observed the high expression of PD-L1 in NII.clusterB-PNI. However, the majority of CIN patients were widely distributed in different subgroups of our classification. This also suggests that our classification method may be useful for these patients in predicting response for immunotherapy. In ACRG classification [52], EMT had the worst prognosis while MSI had the better prognosis than other subtypes.This view is similar to our results in Fig. 1C and Fig. 2D. However, the other two subtypes were more evenly distributed, which also suggests that our classification method is useful in predicting response to immunotherapy, especially for MSS patients in the ACRG classification (Fig. 6C-F).

Based on our analysis in this study, inflammation caused by PNI was varied in GC patients, and the immune cell infiltration, somatic mutation and methylation of subtypes also differed among patients.These findings significantly affect the prognosis and treatment of GC patients.Therefore, we translated the NII cluster system into the NII score system and visualized it as a nomogram with other clinical characteristics.This information is useful for clinicians to calculate a specific score for each patient.The NII score system and nomogram exhibited excellent performance in predicting patient prognosis.We have also demonstrated the stability and efficacy of the system in other tumor types.In addition, the NII scoring system can be used to identify patients who may benefit from immunotherapy and chemotherapy.Compared with other prediction models based on the inflammation induced by PNI [53, 54], we offered a more precise classification of patients.GC patients with PNI and high NII scores may benefit more from immunotherapy and chemotherapy.Other applicable molecular drugs can be identified for other types of patients.

Some limitations in this study should be noted.First, given the influence of inflammation induced by PNI, it was difficult to understand whether the two PNI-related types had opposite developmental patterns or whether NII.clusterA-PNI patients develops into NII.clusterB-PNI patients during PNI progression.More in-depth exploration is needed in the future.However, this phenomenon does not affect the ability of the NII system to judge and predict the immediate state of patients,especially before accepting immunotherapy.When patients choose to receive immunotherapy on the basis of PDL1 expression (CPS score), the prediction of treatment benefit is often imprecise (Fig. 8G). Understanding the regulatory role of the nervous system in the tumor microenvironment may bring more help. Second, the signature gene of NII.clusterB-PNI,VCAM1,is need to explore in the future about the interaction with STAT3 in regulating the expression of PD-L1,which will be a hopeful biomarker to develop new treatments for GC patients.Third, we lacked a large prospective cohort to verify the validity and accuracy of our model's identification and prediction.However, we attempted to address this problem by validating our model with a large retrospective cohort and tissue samples.

In conclusion, we have identified different subtypes of neuroinflammation in GC patients.Based on the features of these subtypes, we developed and validated an NII scoring system and visualized it into a nomogram, which could be used to predict the prognosis, immunotherapy and chemotherapy benefit of GC patients.This instrument represent a potential tool for clinicians in the treatment of GC patients.

### Supplementary Information

**Additional file 1. **

**Additional file 2:** **Figure S1.** Establishment process and distribution of inflammation score. **Figure S2.** Establishment, prognostic significance and distribution of NII.cluster subtypes. **Figure S3.** The noteworthy differentially-expressed genes among NII cluster subtypes. **Figure S4.** The analyse of NII cluster subtypes and non-PNI in mutational signatures, methylation and CNV. **Figure S5.** Verification for prognosis of NII score in pan digestive tract cohorts. **Figure S6.** The verification that NII score serves as an independent prognostic factor using forest plots and subgroup analyses.** Figure S7.** The immune scores, stromal scores, ESTIMATE scores and potential therapeutic chemicals of NII score subgroups. **Figure S8.** The supplementary immunotherapy analyses of NII clusters and non-PNI in NII score subgroups. **Figure S9.** The result of chemosensitivity analyses. **Figure S10.** VCAM1 RNA expression of shRNA and overexpression cell models.

**Additional file 3:** **Table S1.** Clinical and pathologic characteristics of patients with gastric cancer and pan-gastrointestinal tumor. **Table S2.** 200 genes and their functions of MSigdb Hallmarks inflammatory response. **Table S3.** 22 genes from MCODE. **Table S4.** Univariate Cox regreesion of 200 genes with PNI and No PNI. **Table S5.** High NII score VS Low NII score. **Table S6.** Go analysis of No PNI and NII.clusterB. **Table S7.** Description of 47 genes used to construct NII score. **Table S8.** Multivariable Cox regression analysis of variables of nomogram in training cohort and whole cohort. **Table S9.** NII.score. **Table S10.** Subgroup.

## Data Availability

All data generated or analysed during this study are included either in this paper or in the supplementary information. Every reader can obtain the original code of the analysis and the raw data of Nanfang cohorts in this study by contacting us. Expression microarray data of Nanfang cohort 2 can been searched in GEO with GSE214293.
