# Supplementary material for: Predicting response to immunotherapy in gastric cancer via assessing perineural invasion-mediated inflammation in tumor microenvironment
Source: J Exp Clin Cancer Res. 2023 Aug 11;42:206. doi: 10.1186/s13046-023-02730-0 (PMC10416472; doi:10.1186/s13046-023-02730-0)
Supplement: Supplementary file 1 — Additional file 1. [file 13046_2023_2730_MOESM1_ESM.docx]

**Supplementary File**

**Materials and method**

**Data retrieval and preprocessing**

Publicly available transcriptomic cohorts for GC and CRC with clear perineural invasion (PNI) information were systematically searched. PNI annotations were extracted from surgical pathology reports provided by TCGA provisional studies on Cancer Digital Slide Archive (https://cancer.digitalslidearchive.org/), and other clinical information was downloaded from TCGA database. Next, RNA sequencing (Workflow Type: FPKM) and clinical and pathological information were collected from GDC using the R package TCGAbiolinks. HTSeq-FPKM data were transformed into transcripts per million (TPM) reads and then log2 transformed. All raw data and clinical information from microarray cohorts GSE62254 (n=247), GSE15459 (n=192), GSE84437 (n=433), GSE13861 (n=65), GSE26899 (n=93), and GSE26901 (n=109) were downloaded from the GEO database (https://www.ncbi.nlm.nih.gov/geo/). Corresponding PNI features of the GSE62254 cohort were obtained from the supplementary materials in published literature. The comBat algorithm was applied to merge GC patients from the GSE62254 cohort and TCGA datasets enrolled in our study into a multicohort. Nanfang cohort 1 (344 patients with GC diagnosed from October 2004 to September 2011, analyzed for survival prognosis) and Nanfang cohort 2 (27 patients with GC diagnosed from January 2021 to January 2022, before chemotherapy and immunotherapy, RNA-sequencing performed by Shanghai Oebiotech Corporation) were analyzed using standard pathologic criteria at the Southern Medical University Nanfang Hospital.

In this study, the multicohort as the candidate training cohort included 360 patients with clear PNI information. The validation cohorts included 3 independent GC cohorts (GSE15459, GSE84437 and Nanfang cohort 2) and one large pooled cohort ( including 267 patients who underwent gastrectomy as primary treatment in three cohorts (KUGH cohort, GSE26899; KUCM cohort, GSE26901; YUSH cohort, GSE13861)).

Moreover, for further validation, transcriptomic and clinical data of patients with other digestive system tumors, including esophageal cancer, liver cancer, and pancreatic cancer, 1175 patients (GSE39582, GSE17536, TCGA-COAD) with colon cancer, 413 patients (TCGA-LIHC) with liver cancer, and 259 patients (TCGA-PAAD, GSE85916) with pancreatic cancer were downloaded from the GEO and TCGA databases.

**Development and pangastrointestinal neoplasm validation of the Prognostic Inflammatory Response-Related Gene Signature in STAD**

Two hundred inflammatory response-related genes were downloaded from the Molecular Signatures database (MSigDB) and are provided in Table S2. Univariate Cox analysis was performed to identify inflammatory response-related genes with considerable prognostic value in the multicohort. To explore the interactions between inflammatory response-related prognostic genes, we used the STRING database (STRING v10: protein‒protein interaction(PPI) networks, integrated over the tree of life). Cytoscape MCODE (a software environment for integrated models of biomolecular interaction networks) plug-in provided access to select hub modules of the PPI network. The default parameters were as follows: k-core = 2, degree cutoff = 2, max. depth = 100 and node score cutoff = 0.2. Subsequently, LASSO Cox regression analysis based on the package “glmnet” in R was applied to build an optimal prognostic signature closely associated with the inflammatory response in STAD. The Cox regression model with LASSO was used for dimension reduction to reduce noise or redundant genes. The optimal values of the penalty parameter λ were determined through 10 cross-validations. An inflammation score was generated for each patient using the following formula:

Inflammation score = ∑ βi*Expi,

where βi is the coefficient of each gene in the final Cox model and Expi represents the gene expression value.

Then, Kaplan‒Meier survival analysis was used to evaluate the prognostic value of the inflammation score. To validate the independent prognostic ability, multivariate Cox regression analysis was performed, and the results were visualized with the package “forestmodel” in R. GSEA was performed to validate the robust inflammation response status in the high inflammation score group using Gene Set Enrichment Analyses (GSEA) software (Java version 4.0). Moreover, the Wilcoxon test was used to verify the correlation between PNI status and inflammation burden. Similarly, we also calculated the inflammation score of CRC patients using the formula mentioned above and demonstrated the relationship among PNI, inflammation score and patient prognosis.

**Inference of infiltrating cells in the TME**

Gene expression data were employed to characterize the immune tumor microenvironment of samples using a variety of bioinformatics tools. The infiltration level of an immune signature (represented by a set of marker genes) in a sample was quantified by the single-sample gene-set enrichment analysis (ssGSEA) score. The marker gene set for the TME infiltration immune cell type was obtained from Bindea et al[44]. The R package “ESTIMATE” was applied to calculate the immune score, stromal score, and ESTIMATE score. The methods mentioned above were used to confirm the role of the inflammation score in modulating cancer immunity in STAD at the cellular level.

**Identification and consensus clustering of** **neuroinflammatory genes for GC**

To further explore the inflammatory differences within PNI, a more precise classification, or consensus clustering, was performed. Univariate Cox analysis was applied to identify genes associated with both inflammation and prognosis in patients with perineural invasion (26 genes in total). Correlation analysis was performed to clarify the immune characterization of these genes. Pathway analysis was performed to demonstrate the relationship between the genes selected and inflammation. Then, consensus clustering was applied using these genes to identify distinct neuroinflammatory patterns. This procedure was performed using the ConsensusClusterPlus R package and was repeated 1,000 times to ensure the stability of classification. Two subtypes based on inflammation-related gene expression were defined as “NII.clusterA” and “NII.clusterB”. Subsequently, we used PNI and No PNI as labels to perform survival analysis on NII.clusterA and B, and significantly different prognoses were exclusively found in the PNI group. Finally, we divided STAD patients into three subtypes: NII.clusterA-PNI, NII.clusterB-PNI and No PNI.

**Transcriptome analysis among PNI-related subtypes**

Gene Ontology (GO) enrichment analysis, KEGG analysis and Gene Set Enrichment Analysis (GSEA, JAVA version) were used to investigate the differences in signal transduction pathways among the three subtypes. Then, tumor-infiltrating immune cell differences among the three subtypes were assessed using ssGSEA and the Estimate algorithm. Furthermore, we curated a set of gene sets to represent specific biological processes constructed by Mariathasan et al[45]. We collected information on approximately 45 immunomodulators. ANOVA was performed to calculate the relative expression of immune-related genes among the three subtypes. GSVA was also performed using the R package Piano together with the version 7.5.1 annotated gene set (Hallmarks, KEGG) to assess the variation in pathway activity among the three subtypes. Furthermore, we also performed a clinical correlation analysis for the three subtypes using Fisher's exact test.

**Multiomics data analyses**

Differences in somatic mutations, CNVs and DNA methylation among three clusters were performed to comprehend the molecular characterization and differences among these three subtypes. Somatic mutation information was analyzed with MuTect2. The “maftools” package was used to display the high-frequency mutated genes of each subtype in a waterfall plot. In addition, significantly mutated genes (P < 0.05) among the three subtypes and the interaction effect of gene mutations were analyzed by maftools. CNV data were accessed from the Broad Institute STAD FireBrowse. GISTIC 2.0 was applied to identify significantly amplified or deleted genomes, which was a revised computational program to identify somatic copy number alterations by investigating the frequency and amplitude of observed events. Segment mean values greater than 0.2 were defined as a gain, and values less than −0.2 were defined as a loss. Broad values defined by each chromosome arm were analyzed independently. The burden of copy number loss or gain was calculated as the total number of genes with copy number changes at the focal and arm levels. In addition, the Wilcoxon test was used to compare the NII.clusterA-PNI and NII.clusterB-PNI subtypes. For DNA methylation analysis, TCGA gastric adenocarcinoma methylation 450k dataset was downloaded from the UCSC Xena database (https://xena.ucsc.edu/). Data were obtained from the Illumina Infinium Human Methylation 450 Bead Chip platform. The beta matrix was filtered using the ChAMP.filter() function of the ChAMP R package. The filtered matrix was normalized using the default BMIQ method. The promoter of the CpG island is determined by the 450 k annotation file, and its features include TSS200 (the 200-bp upstream region of the transcription site (TSS), 1st Exon (the first exon), TSS1500 (upstream of the TSS-200, an ~1500-bp region) and the 5' UTR. A preliminary screen for differentially methylated CpG sites was performed using the ChAMP R package. CpG sites in this study in accordance with ||>0.2 and Benjamini‒Hochberg adjusted P<0.05 were identified as differentially methylated CpG sites. Differentially methylated CpG sites located in promoter regions (5' UTR, TSS200, TSS1500 and 1st Exon) were further screened and visualized using the pheatmap R package. Tumor-related genes were obtained at https://www.oncokb.org/cancerGenes, and a total of 1066 genes were obtained.

**Dimension Reduction and Generation of the NII Score**

For transformation from qualitative clustering to quantitative models, we developed an NII score system based on NII.cluster. First, the R package “Limma” was applied to identify differentially expressed genes (DEGs) between NII.clusterA and NII.clusterB for STAD by establishing a significance cutoff criteria of p < 0.05 (adjusted) and absolute fold-change > 1.4. Then, among DEGs, we employed Ranger, a weighted version of random forest, to evaluate the importance of each individual DEG with the R package ranger. A weight of 100% was given to each covariate to ensure a 100% chance to be selected in each tree. The variable importance score (VIS) for the DEGs was estimated, and results were ranked in descending order. The sliding window sequential forward feature selection method (SWSFS) was used to identify the top important DEGs. The SWSFS method employs the DEGs of the random forest (RF) model sequentially based on the order of VIS. Then, we plotted the ‘out of bagging (OOB)’ error, which measured the performance of each model consisting of a specific number of DEGs. The top potential DEGs were screened to construct the NII score when the RF model had the lowest error rate.

Furthermore, unsupervised clustering was applied to categorize the patients into Gene.clusterA and Gene.clusterB subtypes. Using the Boruta algorithm, DEGs were positively and negatively correlated with the clusters as NII gene signatures A and B, respectively. We then curated the expression profile of the final determined genes to perform PCA, and both principal components 1 and 2 were extracted and served as the signature score. Finally, we applied the gene expression grade index to define the NII score of each patient:

NII score=$\sum（PCBi-PCAi）$.

**Construction of integrated prognostic models**

Based on the NII score and other clinical features, recursive partitioning analysis (RPA) was performed to construct a decision tree for risk stratification with the R package rpart. Then, a comprehensive model, including NII score, age, sex, TNM stage and PNI, was constructed and visualized with a nomogram using the packages rms, nomogramEx, and regplot in R. In the nomogram scoring system, each variable was matched with a score, and the total score was obtained by adding the scores across all variables of each sample. The calibration of the nomogram was assessed using calibration curves, which can depict the predictive value between the predicted 3- and 5-year survival events and the virtually observed outcomes. The decision curve was applied to evaluate nomogram accuracy. Furthermore, time-dependent receiver operating characteristic analysis for 1-, 3-, and 5-year survival was performed to measure the predictive power using the R package survivalROC. In addition, receiver operating characteristic curve (ROC) analysis was performed to measure and compare the predictive performance of the nomogram score and TNM stage system. The Delong test was used to assess the differences between the two models.

### **TME characteristics, chemotherapy and immunotherapy response prediction of NII score subtypes**

To further characterize the correlation between the NII score and TME, methods for evaluating and quantifying the TME mentioned above were assessed. Then, functional enrichment analysis was executed to demonstrate signaling pathway heterogeneity. We predicted the chemotherapeutic response for each sample based on the largest publicly available pharmacogenomics database (the Genomics of Drug Sensitivity in Cancer (GDSC), https://www.cancerrxgene.org/). OncoPredict was used to compute the IC50 values of commonly administered chemotherapeutic drugs in the meta cohort. We focused on antitumor drugs, such as cisplatin, 5-fluorouracil, oxaliplatin, paclitaxel and docetaxel. The Wilcoxon signed-rank test was adopted to determine whether the difference in IC50 values between the NII high and low subtypes were statistically significant. To further evaluate the differences in the sensitivity of chemotherapy drugs between patients with high and low NII scores, drug sensitivity data of cancer cell lines (CCLs) were obtained from the Cancer Therapeutics Response Portal(CTRP) and Profiling Relative Inhibition Simultaneously in Mixtures(PRISM) Repurposing dataset. The CTRP contains the sensitivity data for 481 compounds in over 835 CCLs, and the PRISM contains the sensitivity data for 1448 compounds in over 482 CCLs. Both datasets provide the area under the dose–response curve (AUC) values as a measure of drug sensitivity to chemotherapy, and lower AUC values indicate increased sensitivity to treatment. K nearest neighbor (k-NN) imputation was applied to impute the missing AUC values. Before imputation, compounds with greater than 20% missing data were excluded. Because the CCLs in both datasets were obtained from the Cancer Cell Line Encyclopedia(CCLE) project, molecular data in CCLE were thus used for subsequent CTRP and PRISM analyses to clarify the sensitivity of chemotherapy in the two subgroups more comprehensively.

**Western blot, immunohistochemical and qPCR analyses**

Western blot: mouse anti-vcam1 antibody (Proteintech,66294-1-Ig), rabbit anti-STAT3 antibody (Abcam, ab68153), rabbit anti-STAT3 (phospho Y705) antibody (Abcam, ab262673), rabbit anti-PDL1 antibody (Abcam, ab205921) and mouse anti-GAPDH antibody (Proteintech,60004-1-Ig).

Immunohistochemical: mouse anti-vcam1 antibody (Proteintech,66294-1-Ig), rabbit anti-STAT3 (phospho Y705) antibody (Abcam, ab262673), mouse anti-S100 antibody (Abcam, ab4066), rabbit anti-wide spectrum cytokeratin antibody (Abcam, ab9377), rabbit anti-CD4 antibody (Abcam, ab133616), rabbit anti-CD8 antibody (Abcam, ab245118), rabbit anti-CD20 antibody (Abcam, ab78237), rabbit anti-CD68 antibody (Abcam, ab283654), rabbit anti-CD28 antibody (Abcam, ab243228), rabbit anti-CD3 antibody (Abcam, ab16669).Multiple fluorescent immunohistochemical: Styramide-Alexa Fluor 546 (AAT, 45025), Styramide-Alexa Fluor 488 (AAT, 45020), Styramide-Alexa Fluor 450(AAT,45010), styramide-Alexa Fluor 514 (AAT, 45022), Styramide-Alexa Fluor 647 (AAT, 1065), and DAPI (Solarbio, C0065).

Primers: VCAM1 (Forward Primer GGGAAGATGGTCGTGATCCTT and Reverse Primer TCTGGGGTGGTCTCGATTTTA), GAPDH (Forward Primer GGAGCGAGATCCCTCCAAAAT and Reverse Primer GGCTGTTGTCATACTTCTCATGG), STAB1(Forward Primer GACTGCCGCTACGAAGTACAG and Reverse Primer CACACACAGGTCCCATTCC),RGS1(Forward Primer TCTTCTCTGCTAACCCAAAGGA and Reverse Primer TGCTTTACAGGGCAAAAGATCAG),P2RX7(Forward Primer TATGAGACGAACAAAGTCACTCG and Reverse Primer GCAAAGCAAACGTAGGAAAAGAT),KCNA3(Forward Primer TTTTCTCCAGCGCGGTCTAC and Reverse Primer CATATCGCCGTAACCCACTGT),IL12B(Forward Primer ACCCTGACCATCCAAGTCAAA and Reverse Primer TTGGCCTCGCATCTTAGAAAG),IL10RA(Forward Primer CCTCCGTCTGTGTGGTTTGAA and Reverse Primer CACTGCGGTAAGGTCATAGGA),EBI3(Forward Primer TCATTGCCACGTACAGGCTC and Reverse Primer GGGTCGGGCTTGATGATGTG),NGFR(Forward Primer CCTACGGCTACTACCAGGATG and Reverse Primer CACACGGTGTTCTGCTTGT),BDNF(Forward Primer GGCTTGACATCATTGGCTGAC and Reverse Primer CATTGGGCCGAACTTTCTGGT),GDNF(Forward Primer GGCAGTGCTTCCTAGAAGAGA and Reverse Primer AAGACACAACCCCGGTTTTTG),NTF3(Forward Primer GAACTGCTGCGACAACAGAGA and Reverse Primer CCCACGTAATCCTCCATGAGA),NCAM1(Forward Primer GGCATTTACAAGTGTGTGGTTAC and Reverse Primer TTGGCGCATTCTTGAACATGA),DCLK1(Forward Primer ACTTCGACGAGCGGGATAAG and Reverse Primer GGGCCTCAAAAGATCGGAACC),PDL1(Forward Primer TGGCATTTGCTGAACGCATTT and Reverse Primer TGCAGCCAGGTCTAATTGTTTT),TGFB1(Forward Primer GGCCAGATCCTGTCCAAGC and Reverse Primer GTGGGTTTCCACCATTAGCAC),BTLA(Forward Primer CATCTTAGCAGGAGATCCCTTTG and Reverse Primer GACCCATTGTCATTAGGAAGCA),LAG3(Forward Primer GCGGGGACTTCTCGCTATG and Reverse Primer GGCTCTGAGAGATCCTGGGG),HAVCR2(Forward Primer CTGCTGCTACTACTTACAAGGTC and Reverse Primer GCAGGGCAGATAGGCATTCT),IDO1(Forward Primer GCCAGCTTCGAGAAAGAGTTG and Reverse Primer ATCCCAGAACTAGACGTGCAA),TIGIT(Forward Primer TCTGCATCTATCACACCTACCC and Reverse Primer CCACCACGATGACTGCTGT).
