# Supplementary material for: Predicting response to immunotherapy in gastric cancer via assessing perineural invasion-mediated inflammation in tumor microenvironment
Source: J Exp Clin Cancer Res. 2023 Aug 11;42:206. doi: 10.1186/s13046-023-02730-0 (PMC10416472; doi:10.1186/s13046-023-02730-0)
Supplement: Supplementary file 2 — Additional file 2: Figure S1. Establishment process and distribution of inflammation score. Figure S2. Establishment, prognostic significance and distribution of NII.cluster subtypes. Figure S3. The noteworthy differentially-expressed genes among NII cluster subtypes. Figure S4. The analyse of NII cluster subtypes and non-PNI in mutational signatures, methylation and CNV. Figure S5. Verification for prognosis of NII score in pan digestive tract cohorts. Figure S6. The verification that NII score serves as an independent prognostic factor using forest plots and subgroup analyses. Figure S7. The immune scores, stromal scores, ESTIMATE scores and potential therapeutic chemicals of NII score subgroups. Figure S8. The supplementary immunotherapy analyses of NII clusters and non-PNI in NII score subgroups. Figure S9. The result of chemosensitivity analyses. Figure S10. VCAM1 RNA expression of shRNA and overexpression cell models. [file 13046_2023_2730_MOESM2_ESM.zip › Figure S2.pdf]

FIGURE.S2

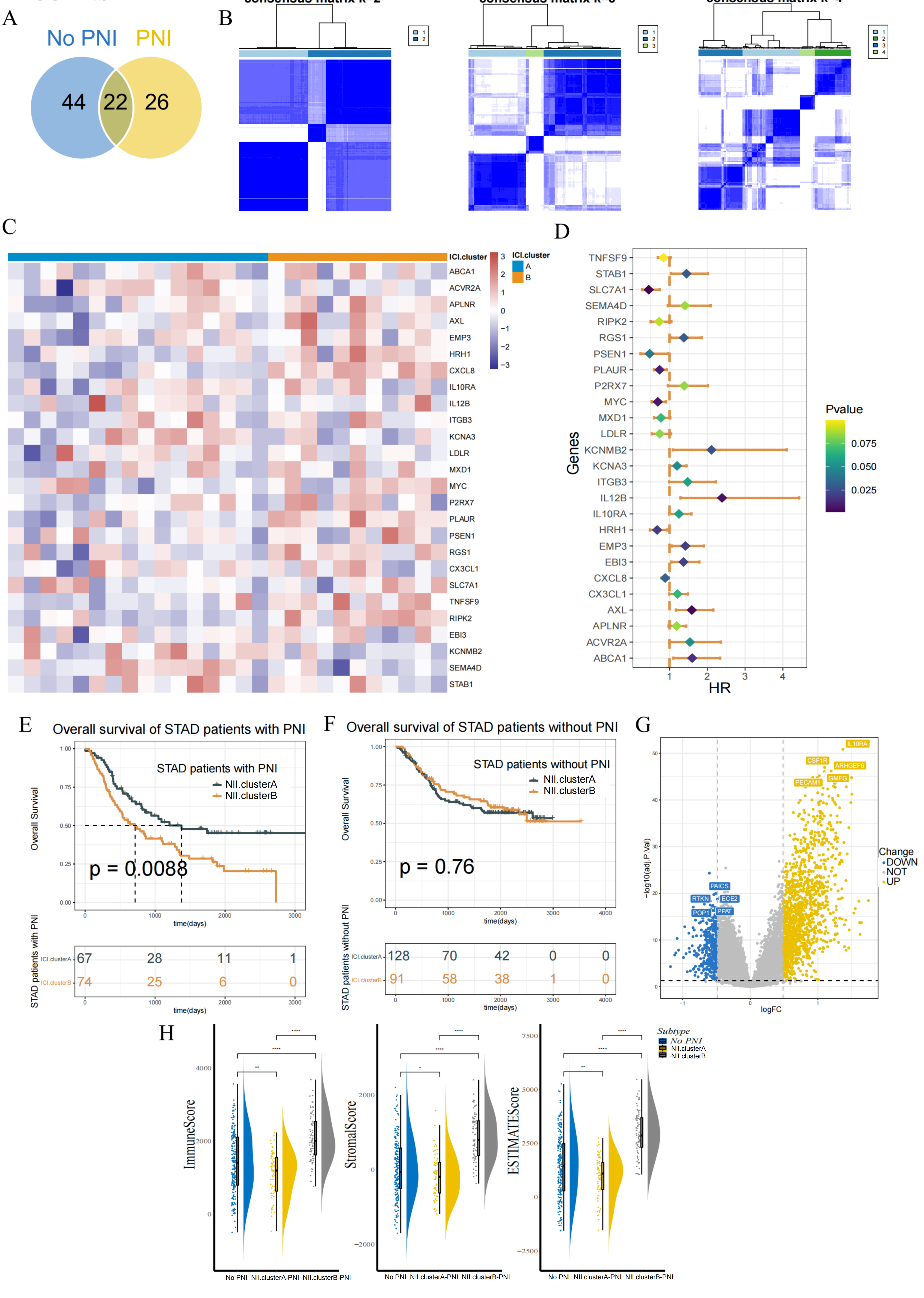
A
No PNI
PNI
44
22
26
B
consensus matrix k=2
consensus matrix k=3
consensus matrix k=4
C
ICI.cluster
A
B
ABCA1
ACVR2A
APLNR
AXL
EMP3
HRH1
CXCL8
IL10RA
IL12B
ITGB3
KCNA3
LDLR
MXD1
MYC
P2RX7
PLAUR
PSEN1
RGS1
CX3CL1
SLC7A1
TNFSF9
RIPK2
EBI3
KCNMB2
SEMA4D
STAB1
D
Genes
HR
Pvalue
0.075
0.050
0.025
E
Overall survival of STAD patients with PNI
STAD patients with PNI
NII.clusterA
NII.clusterB
p = 0.0088
Overall Survival
time(days)
67
28
11
1
74
25
6
0
F
Overall survival of STAD patients without PNI
STAD patients without PNI
NII.clusterA
NII.clusterB
p = 0.76
128
70
42
0
0
91
58
38
1
0
G
IL10RA
CSF1R
ARHGEF6
GMFG
PECAM1
PAICS
RTKN
ECE2
POP1
PPAT
Change
DOWN
NOT
UP
-log10(adj.P.Val)
logFC
H
Subtype
No PNI
NII.clusterA
NII.clusterB
ImmuneScore
StromalScore
ESTIMATEScore
No PNI
NII.clusterA-PNI
NII.clusterB-PNI
