# Supplementary material for: Predicting response to immunotherapy in gastric cancer via assessing perineural invasion-mediated inflammation in tumor microenvironment
Source: J Exp Clin Cancer Res. 2023 Aug 11;42:206. doi: 10.1186/s13046-023-02730-0 (PMC10416472; doi:10.1186/s13046-023-02730-0)
Supplement: Supplementary file 2 — Additional file 2: Figure S1. Establishment process and distribution of inflammation score. Figure S2. Establishment, prognostic significance and distribution of NII.cluster subtypes. Figure S3. The noteworthy differentially-expressed genes among NII cluster subtypes. Figure S4. The analyse of NII cluster subtypes and non-PNI in mutational signatures, methylation and CNV. Figure S5. Verification for prognosis of NII score in pan digestive tract cohorts. Figure S6. The verification that NII score serves as an independent prognostic factor using forest plots and subgroup analyses. Figure S7. The immune scores, stromal scores, ESTIMATE scores and potential therapeutic chemicals of NII score subgroups. Figure S8. The supplementary immunotherapy analyses of NII clusters and non-PNI in NII score subgroups. Figure S9. The result of chemosensitivity analyses. Figure S10. VCAM1 RNA expression of shRNA and overexpression cell models. [file 13046_2023_2730_MOESM2_ESM.zip › Figure S6.pdf]

FIGURE.S6

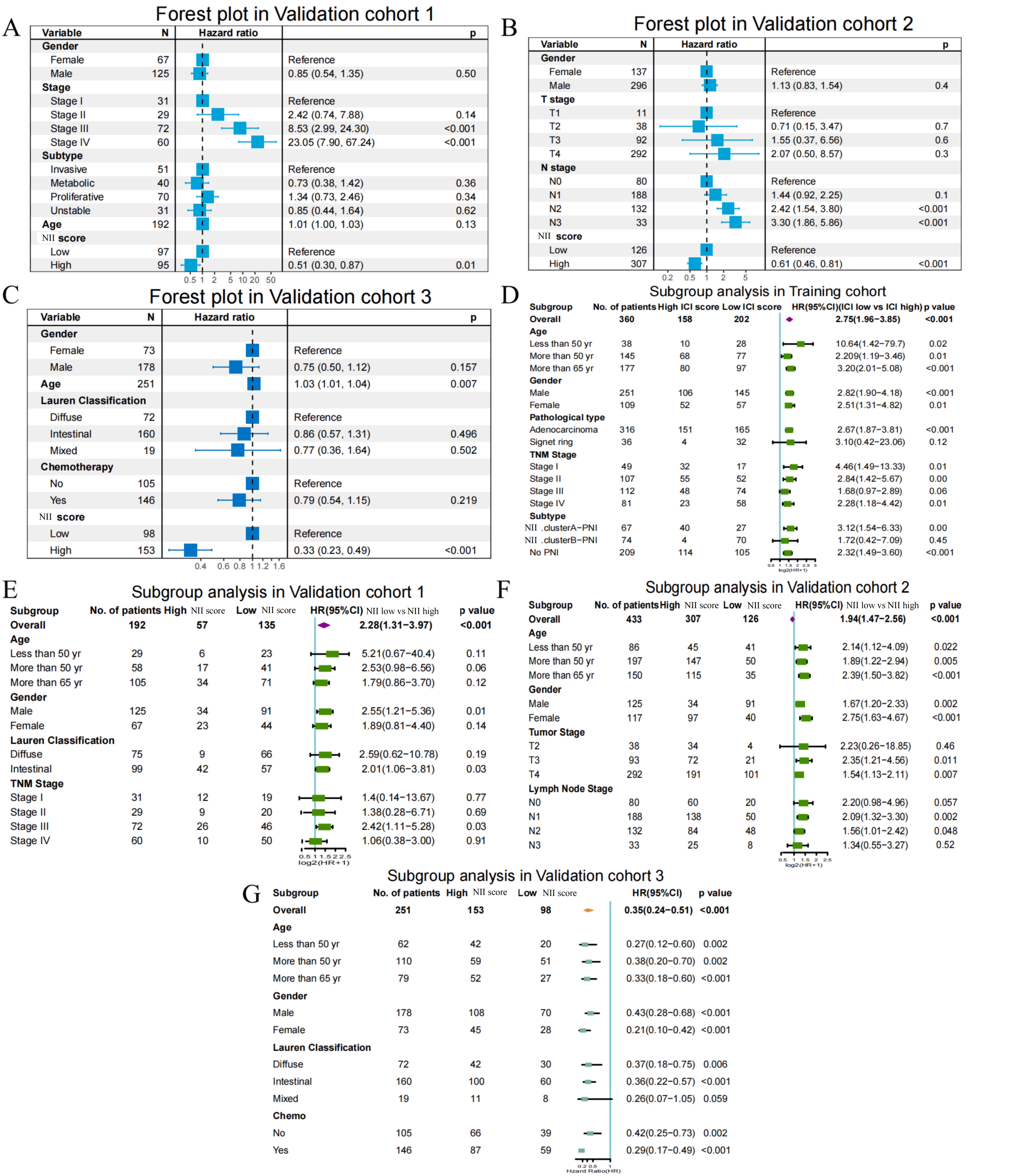
A
Forest plot in Validation cohort 1
Variable N Hazard ratio p
Gender
Female 67 Reference
Male 125 0.85 (0.54, 1.35) 0.50
Stage
Stage I 31 Reference
Stage II 29 2.42 (0.74, 7.88) 0.14
Stage III 72 8.53 (2.99, 24.30) <0.001
Stage IV 60 23.05 (7.90, 67.24) <0.001
Subtype
Invasive 51 Reference
Metabolic 40 0.73 (0.38, 1.42) 0.36
Proliferative 70 1.34 (0.73, 2.46) 0.34
Unstable 31 0.85 (0.44, 1.64) 0.62
Age 192 1.01 (1.00, 1.03) 0.13
NII score
Low 97 Reference
High 95 0.51 (0.30, 0.87) 0.01
0.5 1 2 5 10 20 50
B
Forest plot in Validation cohort 2
Variable N Hazard ratio p
Gender
Female 137 Reference
Male 296 1.13 (0.83, 1.54) 0.4
T stage
T1 11 Reference
T2 38 0.71 (0.15, 3.47) 0.7
T3 92 1.55 (0.37, 6.56) 0.6
T4 292 2.07 (0.50, 8.57) 0.3
N stage
N0 80 Reference
N1 188 1.44 (0.92, 2.25) 0.1
N2 132 2.42 (1.54, 3.80) <0.001
N3 33 3.30 (1.86, 5.86) <0.001
NII score
Low 126 Reference
High 307 0.61 (0.46, 0.81) <0.001
0.2 0.5 1 2 5
C
Forest plot in Validation cohort 3
Variable N Hazard ratio p
Gender
Female 73 Reference
Male 178 0.75 (0.50, 1.12) 0.157
Age 251 1.03 (1.01, 1.04) 0.007
Lauren Classification
Diffuse 72 Reference
Intestinal 160 0.86 (0.57, 1.31) 0.496
Mixed 19 0.77 (0.36, 1.64) 0.502
Chemotherapy
No 105 Reference
Yes 146 0.79 (0.54, 1.15) 0.219
NII score
Low 98 Reference
High 153 0.33 (0.23, 0.49) <0.001
0.4 0.6 0.8 1 1.2 1.6
D
Subgroup analysis in Training cohort
Subgroup No. of patients High ICI score Low ICI score HR(95%CI)(ICI low vs ICI high) p value
Overall 360 158 202 2.75(1.96−3.85) <0.001
Age
Less than 50 yr 38 10 28 10.64(1.42−79.7) 0.02
More than 50 yr 145 68 77 2.209(1.19−3.46) 0.01
More than 65 yr 177 80 97 3.20(2.01−5.08) <0.001
Gender
Male 251 106 145 2.82(1.90−4.18) <0.001
Female 109 52 57 2.51(1.31−4.82) 0.01
Pathological type
Adenocarcinoma 316 151 165 2.67(1.87−3.81) <0.001
Signet ring 36 4 32 3.10(0.42−23.06) 0.12
TNM Stage
Stage I 49 32 17 4.46(1.49−13.33) 0.01
Stage II 107 55 52 2.84(1.42−5.67) 0.00
Stage III 112 48 74 1.68(0.97−2.89) 0.06
Stage IV 81 23 58 2.28(1.18−4.42) 0.01
Subtype
NII .clusterA-PNI 67 40 27 3.12(1.54−6.33) 0.00
NII .clusterB-PNI 74 4 70 1.72(0.42−7.09) 0.45
No PNI 209 114 105 2.32(1.49−3.60) <0.001
log2(HR+1)
E
Subgroup analysis in Validation cohort 1
Subgroup No. of patients High NII score Low NII score HR(95%CI) NII low vs NII high p value
Overall 192 57 135 2.28(1.31−3.97) <0.001
Age
Less than 50 yr 29 6 23 5.21(0.67−40.4) 0.11
More than 50 yr 58 17 41 2.53(0.98−6.56) 0.06
More than 65 yr 105 34 71 1.79(0.86−3.70) 0.12
Gender
Male 125 34 91 2.55(1.21−5.36) 0.01
Female 67 23 44 1.89(0.81−4.40) 0.14
Lauren Classification
Diffuse 75 9 66 2.59(0.62−10.78) 0.19
Intestinal 99 42 57 2.01(1.06−3.81) 0.03
TNM Stage
Stage I 31 12 19 1.4(0.14−13.67) 0.77
Stage II 29 9 20 1.38(0.28−6.71) 0.69
Stage III 72 26 46 2.42(1.11−5.28) 0.03
Stage IV 60 10 50 1.06(0.38−3.00) 0.91
0.5 1 1.5 2 2.5
log2(HR+1)
F
Subgroup analysis in Validation cohort 2
Subgroup No. of patients High NII score Low NII score HR(95%CI) NII low vs NII high p value
Overall 433 307 126 1.94(1.47−2.56) <0.001
Age
Less than 50 yr 86 45 41 2.14(1.12−4.09) 0.022
More than 50 yr 197 147 50 1.89(1.22−2.94) 0.005
More than 65 yr 150 115 35 2.39(1.50−3.82) <0.001
Gender
Male 125 34 91 1.67(1.20−2.33) 0.002
Female 117 97 40 2.75(1.63−4.67) <0.001
Tumor Stage
T2 38 34 4 2.23(0.26−18.85) 0.46
T3 93 72 21 2.35(1.21−4.56) 0.011
T4 292 191 101 1.54(1.13−2.11) 0.007
Lymph Node Stage
N0 80 60 20 2.20(0.98−4.96) 0.057
N1 188 138 50 2.09(1.32−3.30) 0.002
N2 132 84 48 1.56(1.01−2.42) 0.048
N3 33 25 8 1.34(0.55−3.27) 0.52
0.5 1 1.5 2 2.5
log2(HR+1)
G
Subgroup analysis in Validation cohort 3
Subgroup No. of patients High NII score Low NII score HR(95%CI) p value
Overall 251 153 98 0.35(0.24−0.51) <0.001
Age
Less than 50 yr 62 42 20 0.27(0.12−0.60) 0.002
More than 50 yr 110 59 51 0.38(0.20−0.70) 0.002
More than 65 yr 79 52 27 0.33(0.18−0.60) <0.001
Gender
Male 178 108 70 0.43(0.28−0.68) <0.001
Female 73 45 28 0.21(0.10−0.42) <0.001
Lauren Classification
Diffuse 72 42 30 0.37(0.18−0.75) 0.006
Intestinal 160 100 60 0.36(0.22−0.57) <0.001
Mixed 19 11 8 0.26(0.07−1.05) 0.059
Chemo
No 105 66 39 0.42(0.25−0.73) 0.002
Yes 146 87 59 0.29(0.17−0.49) <0.001
0.2 0.5 1
Hazard Ratio(HR)
