# Supplementary figures and images for: Predicting response to immunotherapy in gastric cancer via assessing perineural invasion-mediated inflammation in tumor microenvironment
Source: J Exp Clin Cancer Res. 2023 Aug 11;42:206. doi: 10.1186/s13046-023-02730-0 (PMC10416472; doi:10.1186/s13046-023-02730-0)

# FIGURE.S1

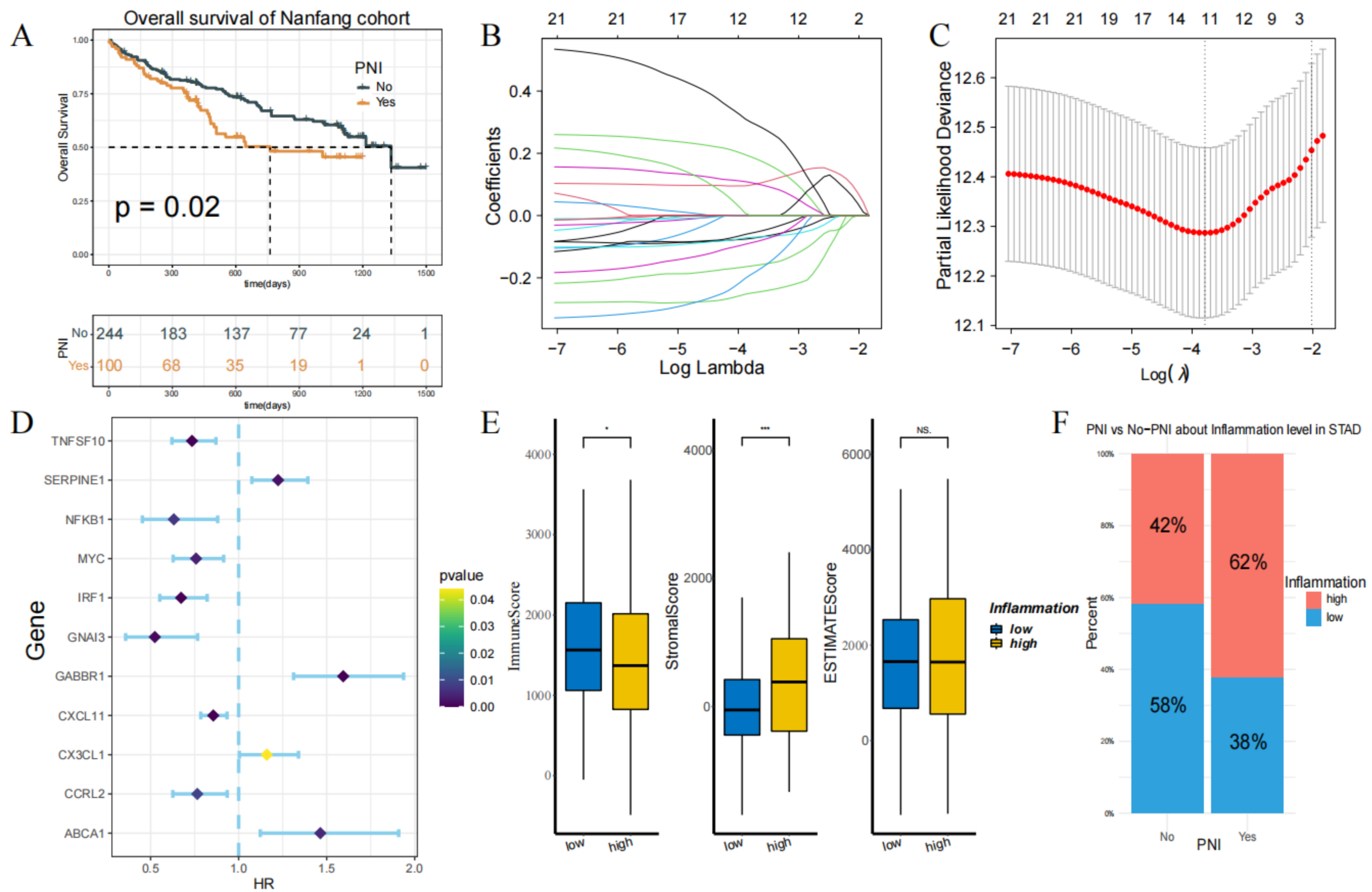

Supplement: Supplementary file 2 — Additional file 2: Figure S1. Establishment process and distribution of inflammation score. Figure S2. Establishment, prognostic significance and distribution of NII.cluster subtypes. Figure S3. The noteworthy differentially-expressed genes among NII cluster subtypes. Figure S4. The analyse of NII cluster subtypes and non-PNI in mutational signatures, methylation and CNV. Figure S5. Verification for prognosis of NII score in pan digestive tract cohorts. Figure S6. The verification that NII score serves as an independent prognostic factor using forest plots and subgroup analyses. Figure S7. The immune scores, stromal scores, ESTIMATE scores and potential therapeutic chemicals of NII score subgroups. Figure S8. The supplementary immunotherapy analyses of NII clusters and non-PNI in NII score subgroups. Figure S9. The result of chemosensitivity analyses. Figure S10. VCAM1 RNA expression of shRNA and overexpression cell models. [file 13046_2023_2730_MOESM2_ESM.zip › Figure S1.pdf]

FIGURE.S10

A

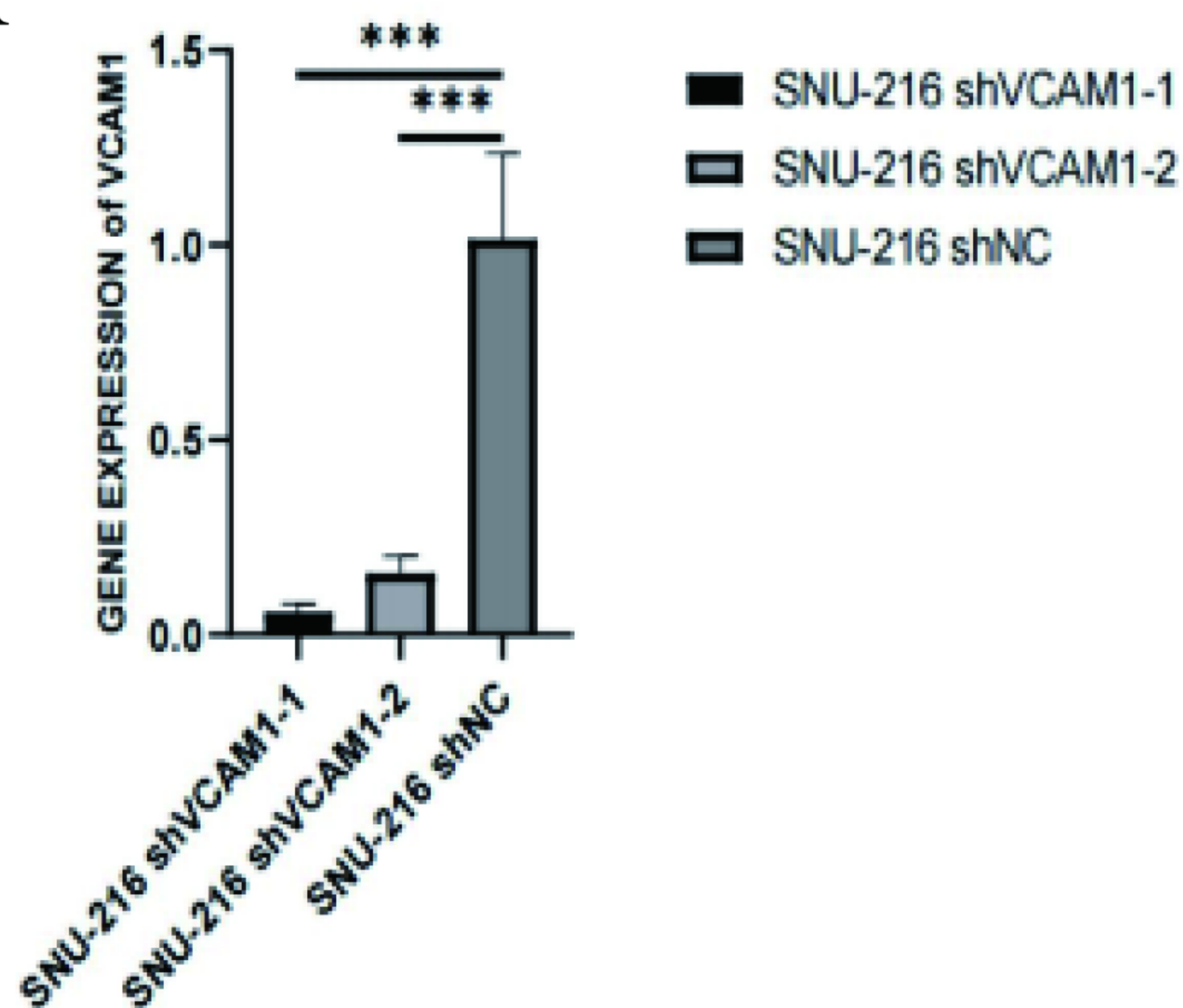

B

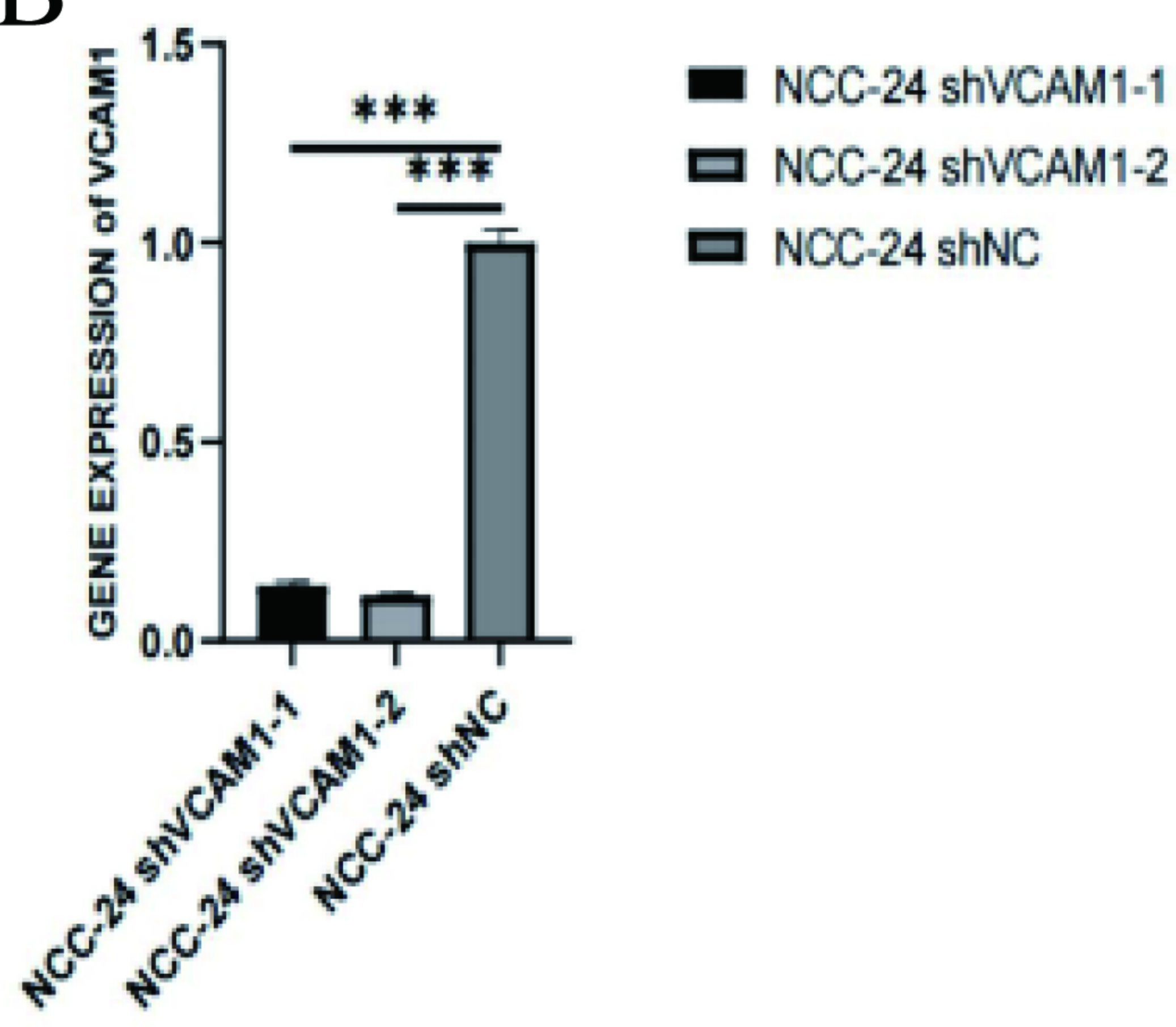

C

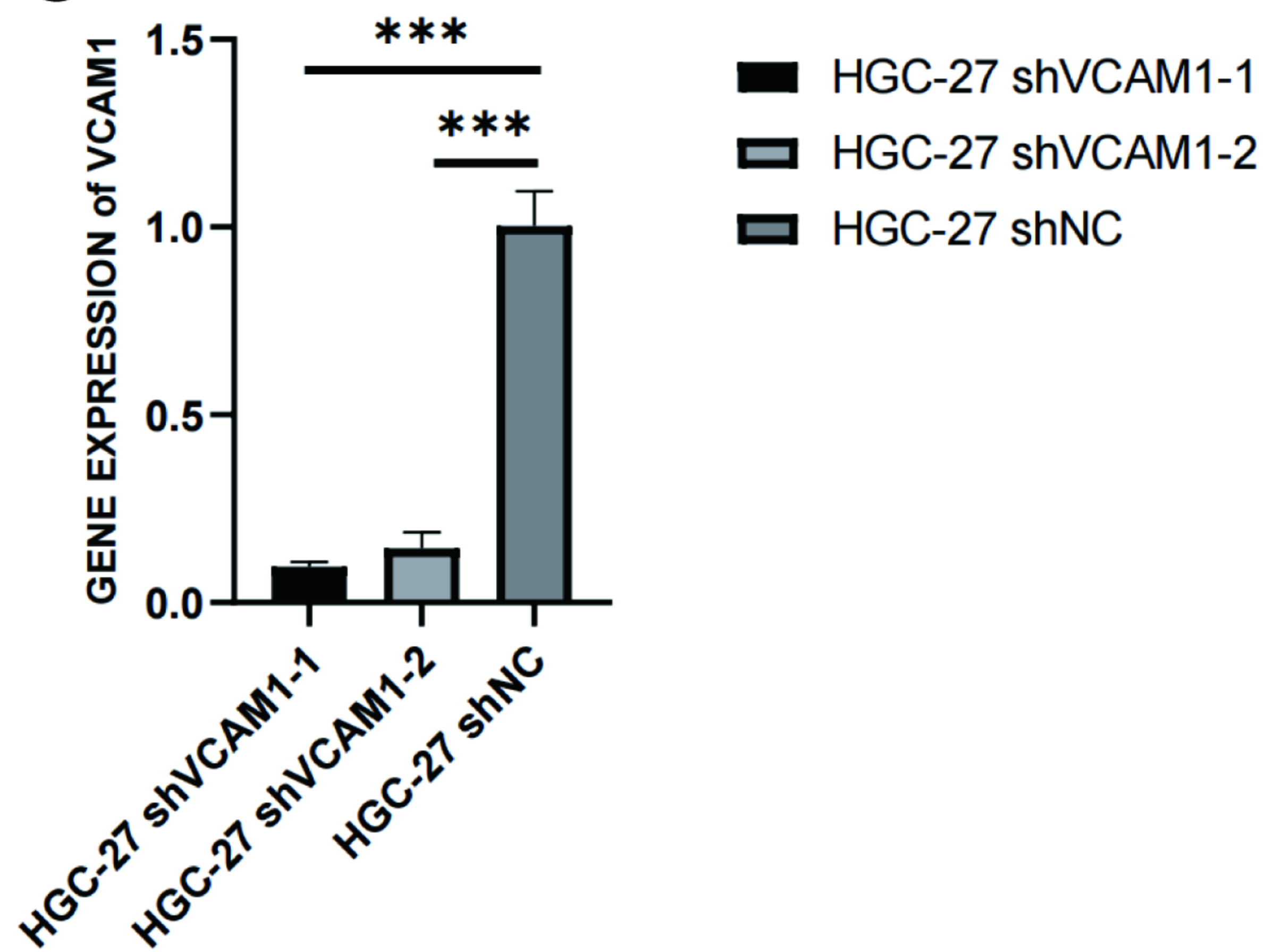

D

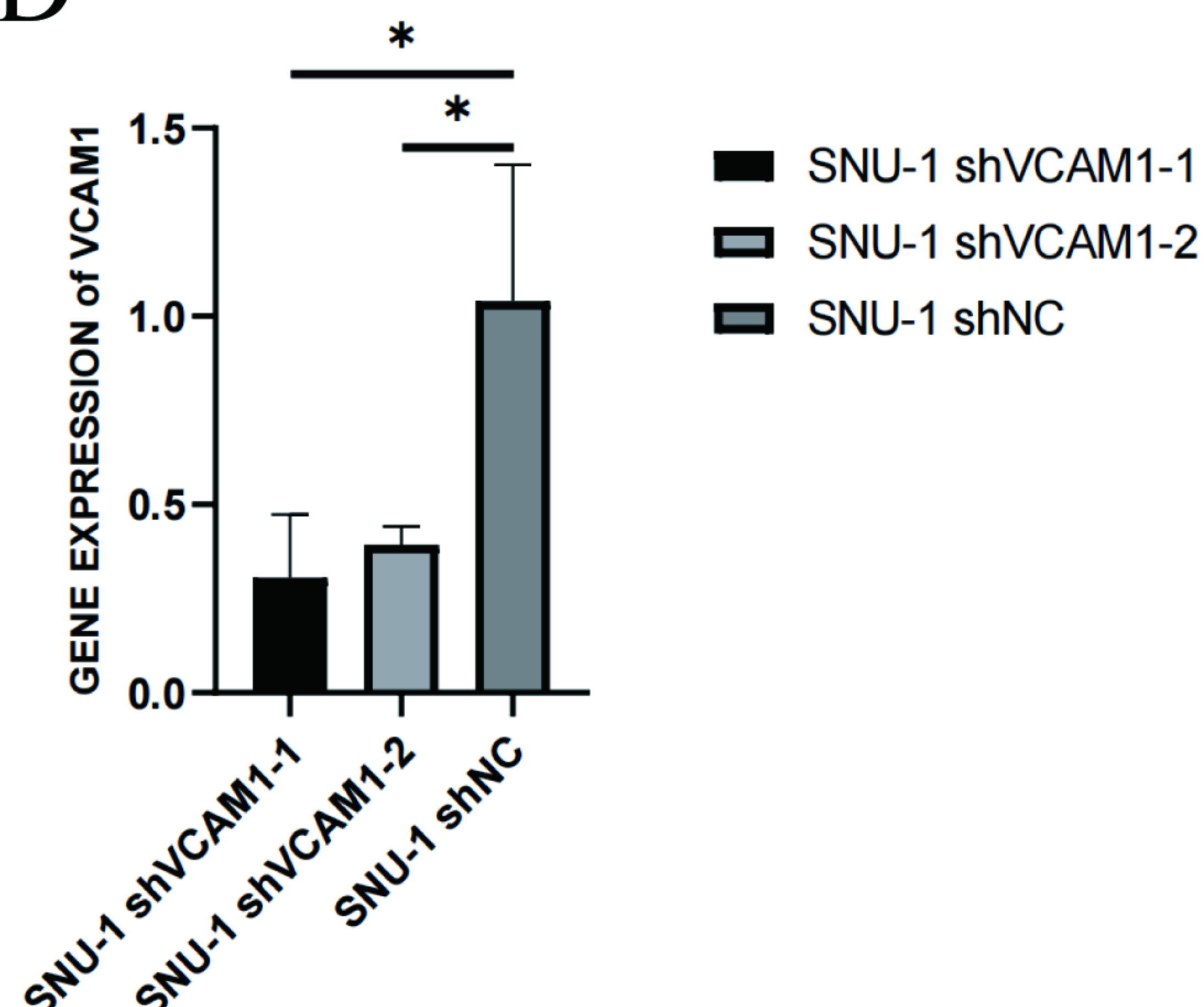

E

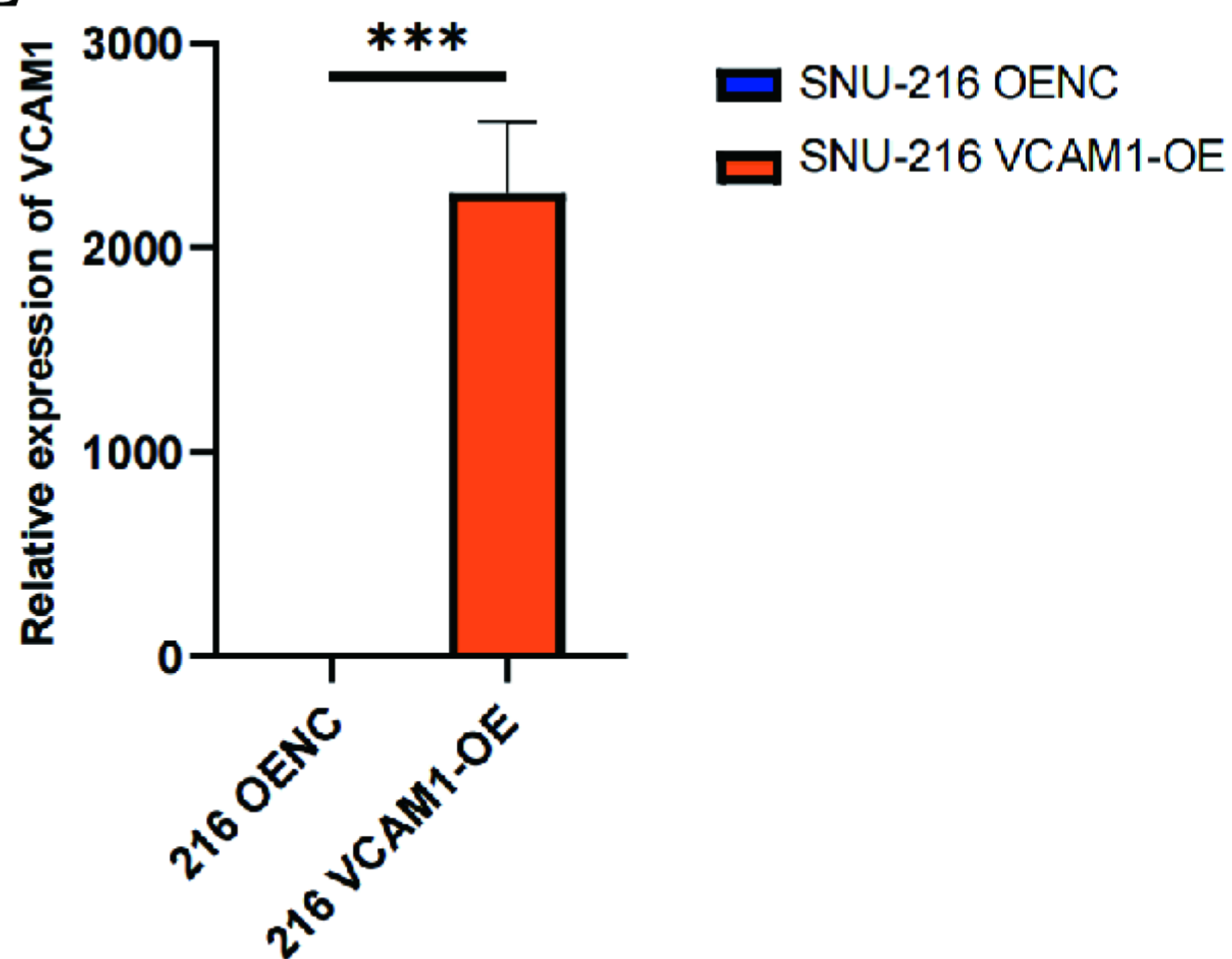

Supplement: Supplementary file 2 — Additional file 2: Figure S1. Establishment process and distribution of inflammation score. Figure S2. Establishment, prognostic significance and distribution of NII.cluster subtypes. Figure S3. The noteworthy differentially-expressed genes among NII cluster subtypes. Figure S4. The analyse of NII cluster subtypes and non-PNI in mutational signatures, methylation and CNV. Figure S5. Verification for prognosis of NII score in pan digestive tract cohorts. Figure S6. The verification that NII score serves as an independent prognostic factor using forest plots and subgroup analyses. Figure S7. The immune scores, stromal scores, ESTIMATE scores and potential therapeutic chemicals of NII score subgroups. Figure S8. The supplementary immunotherapy analyses of NII clusters and non-PNI in NII score subgroups. Figure S9. The result of chemosensitivity analyses. Figure S10. VCAM1 RNA expression of shRNA and overexpression cell models. [file 13046_2023_2730_MOESM2_ESM.zip › Figure S10.pdf]

FIGURE.S4

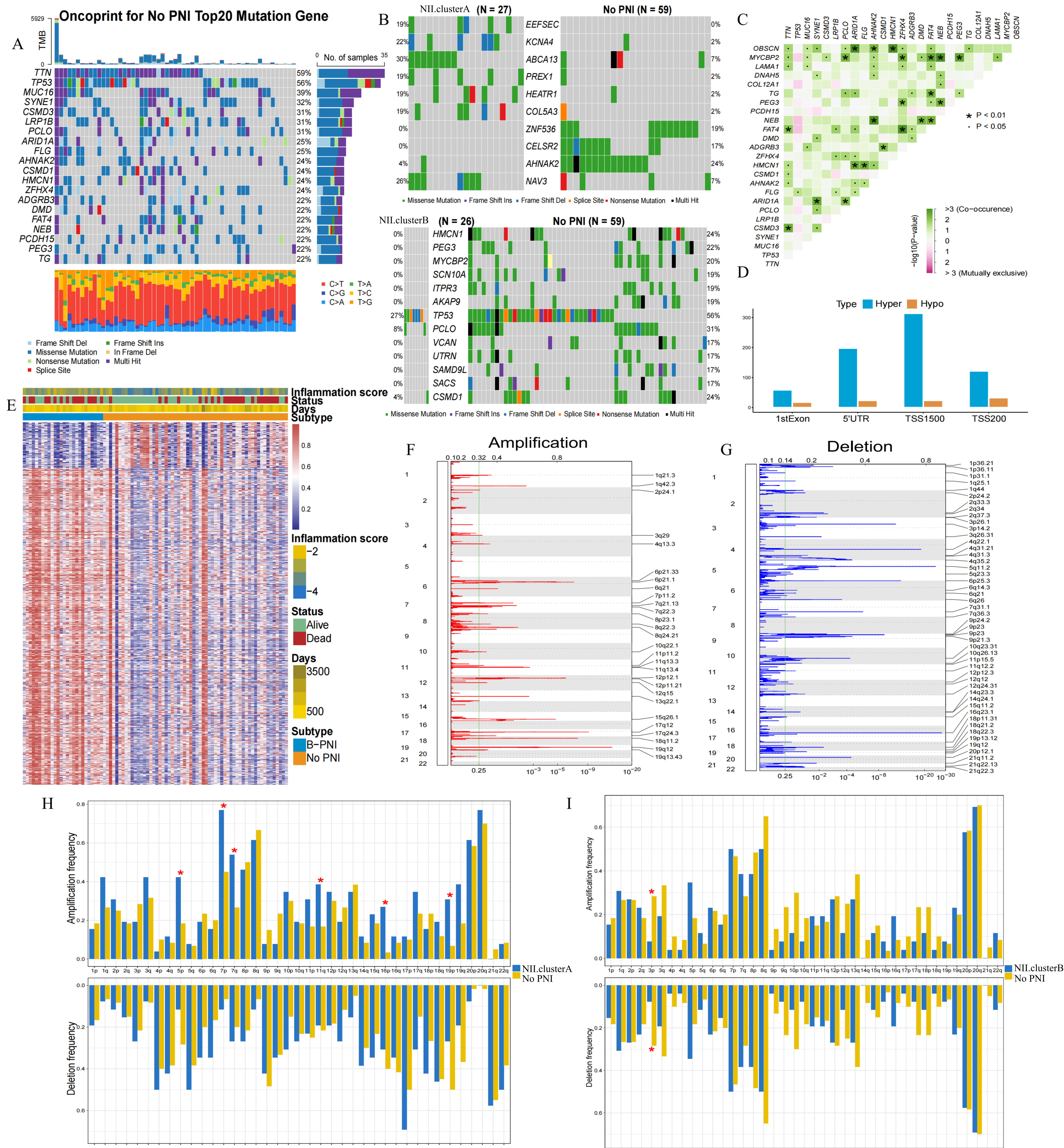

Supplement: Supplementary file 2 — Additional file 2: Figure S1. Establishment process and distribution of inflammation score. Figure S2. Establishment, prognostic significance and distribution of NII.cluster subtypes. Figure S3. The noteworthy differentially-expressed genes among NII cluster subtypes. Figure S4. The analyse of NII cluster subtypes and non-PNI in mutational signatures, methylation and CNV. Figure S5. Verification for prognosis of NII score in pan digestive tract cohorts. Figure S6. The verification that NII score serves as an independent prognostic factor using forest plots and subgroup analyses. Figure S7. The immune scores, stromal scores, ESTIMATE scores and potential therapeutic chemicals of NII score subgroups. Figure S8. The supplementary immunotherapy analyses of NII clusters and non-PNI in NII score subgroups. Figure S9. The result of chemosensitivity analyses. Figure S10. VCAM1 RNA expression of shRNA and overexpression cell models. [file 13046_2023_2730_MOESM2_ESM.zip › Figure S4.pdf]

FIGURE.S5

A

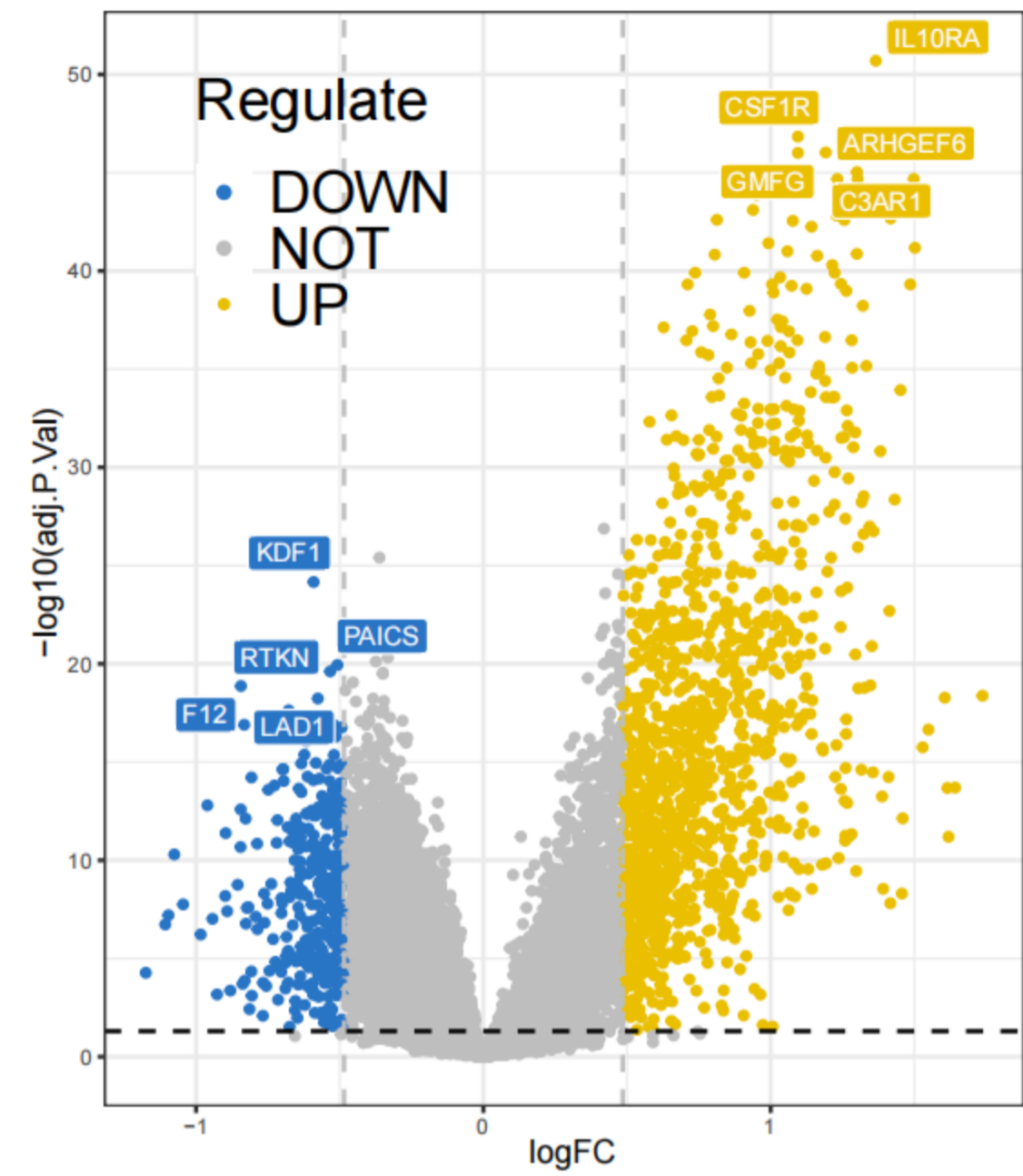

B

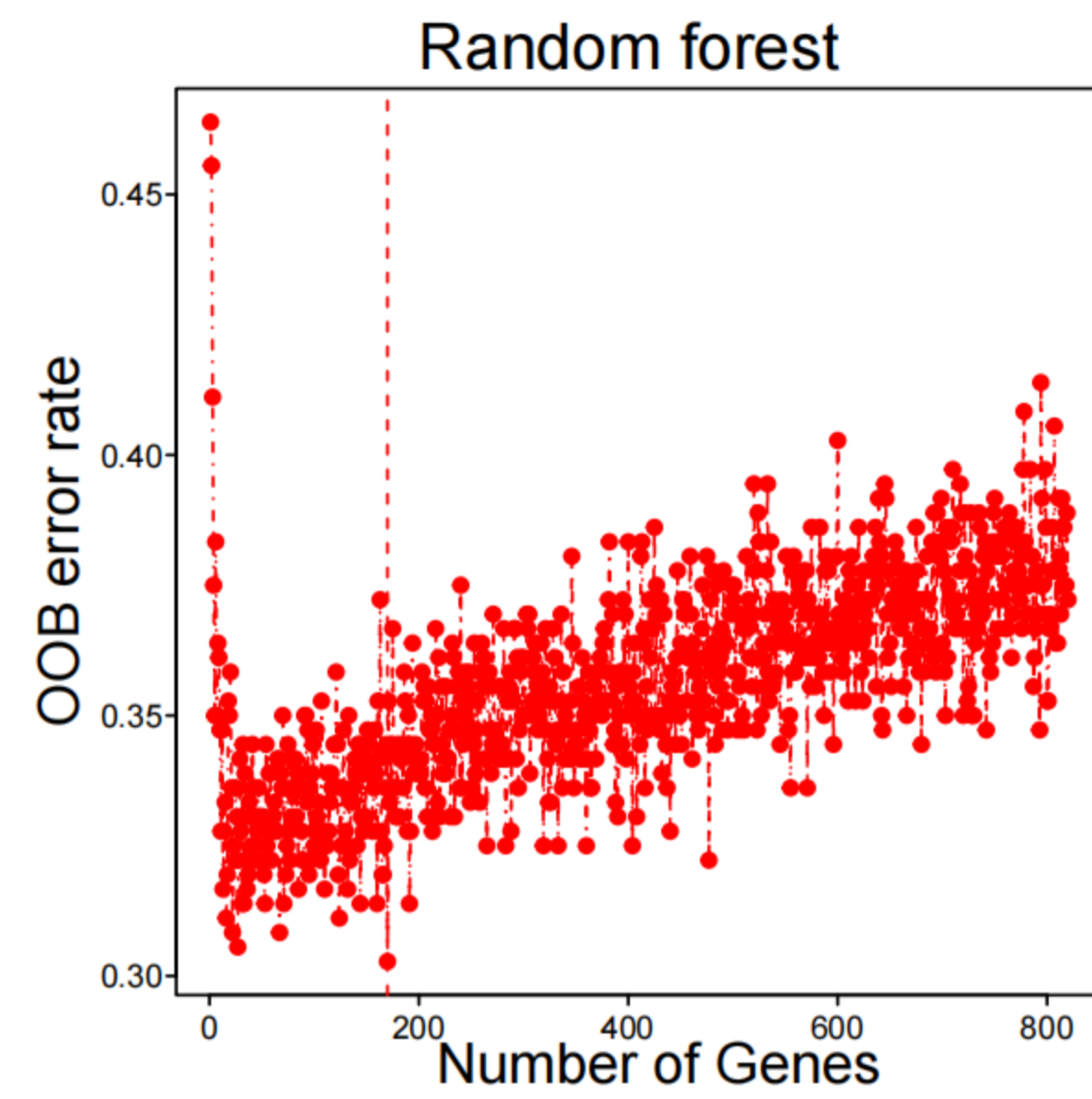

C

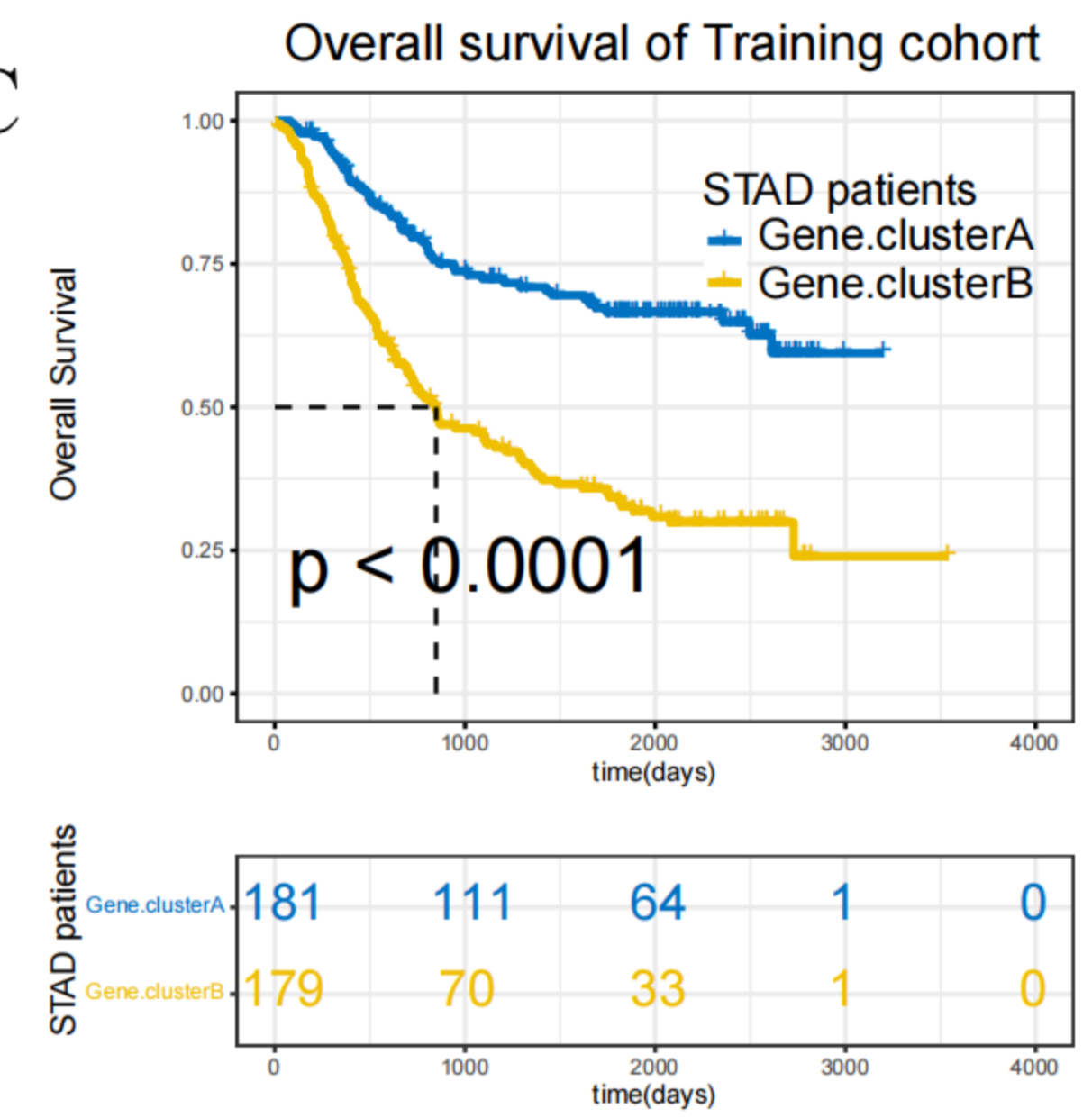

D

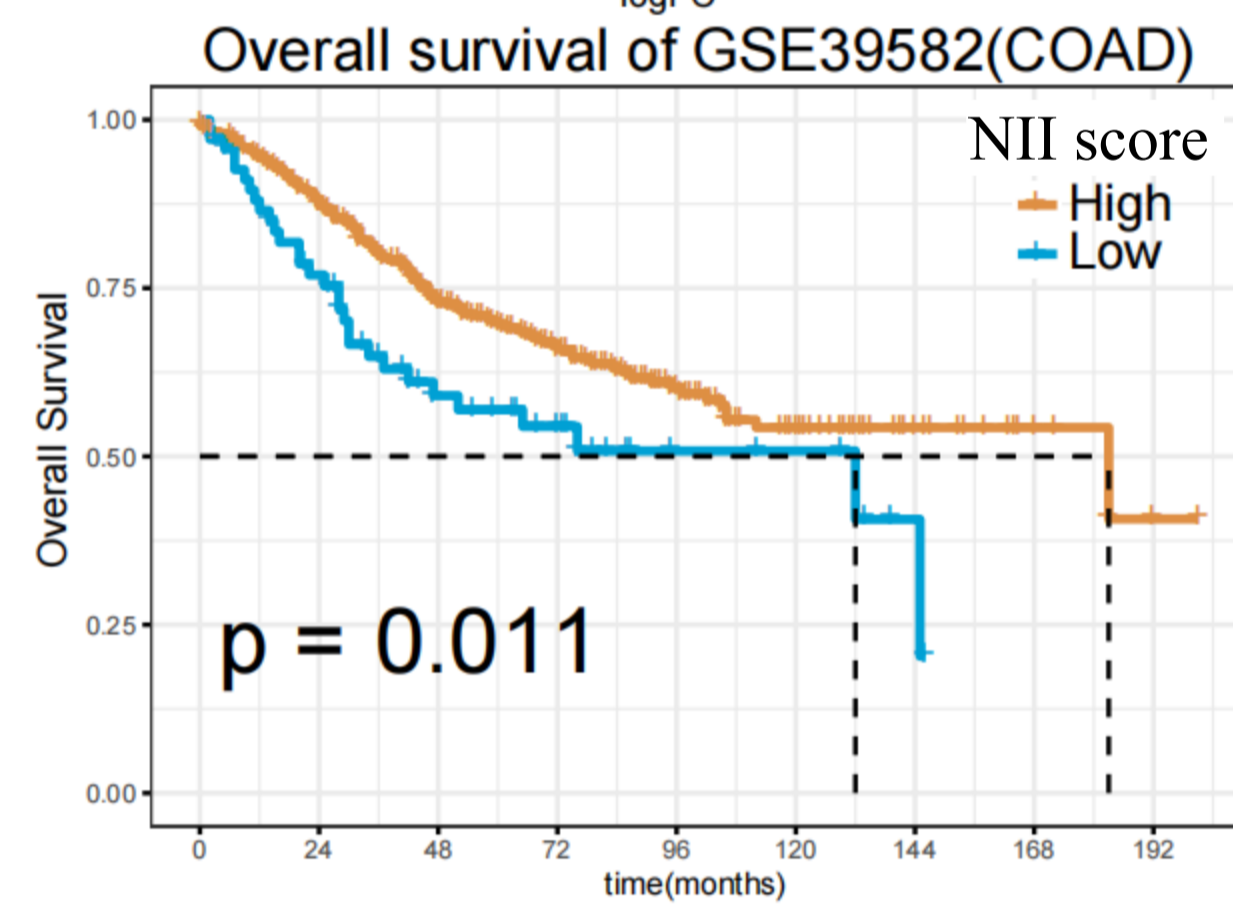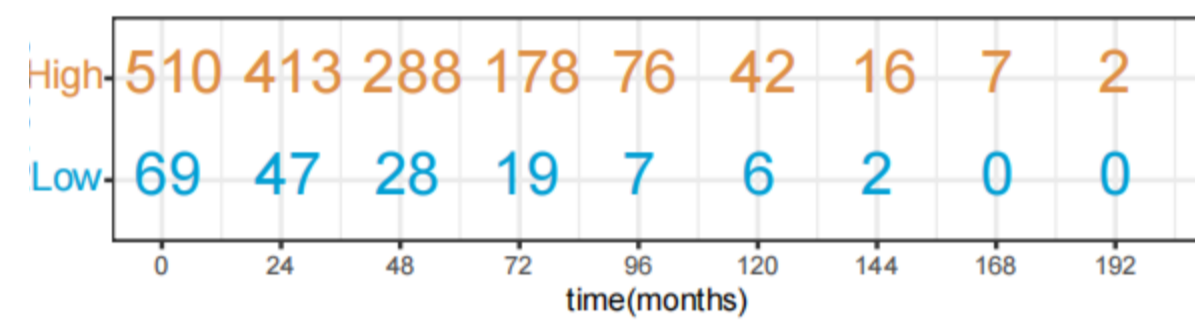

E

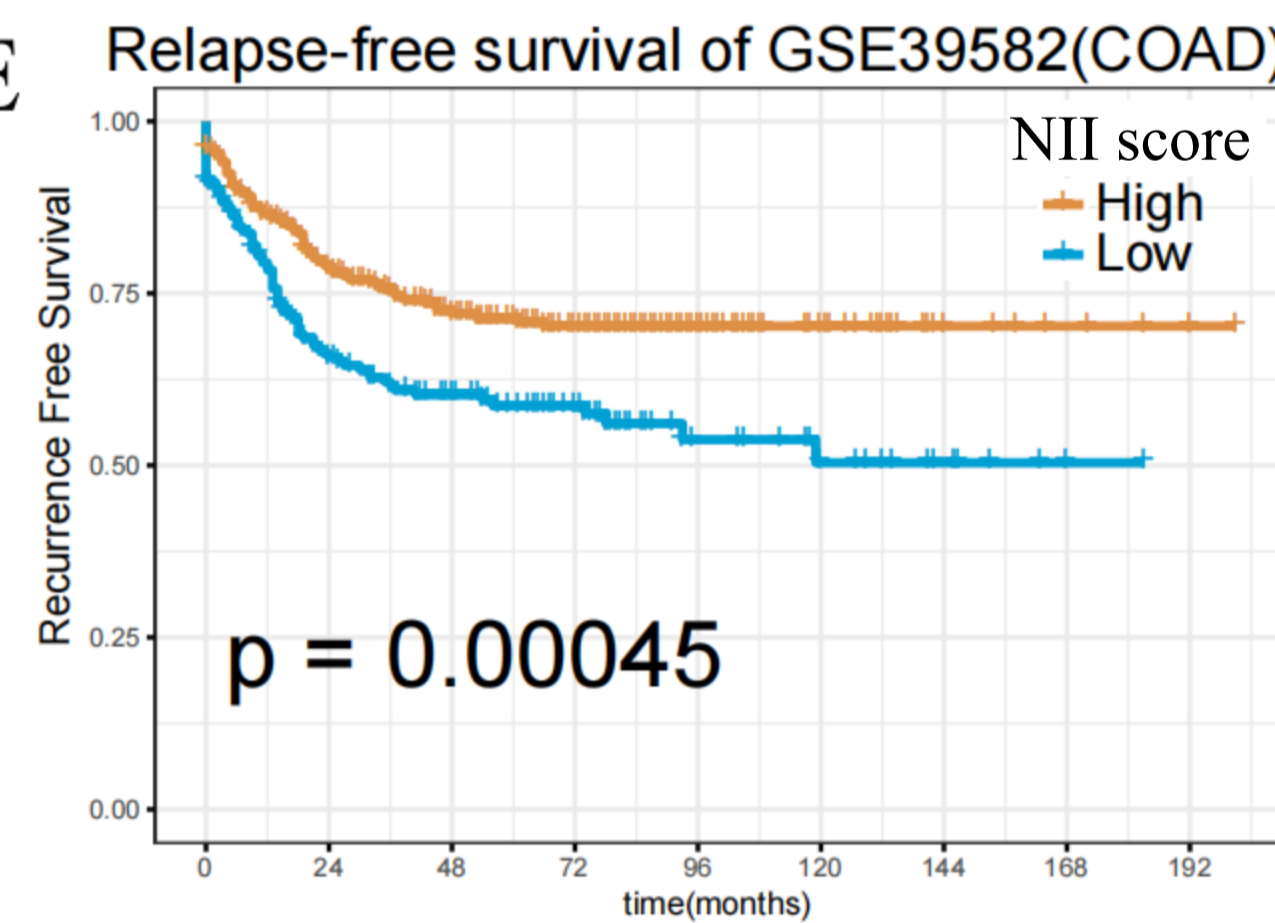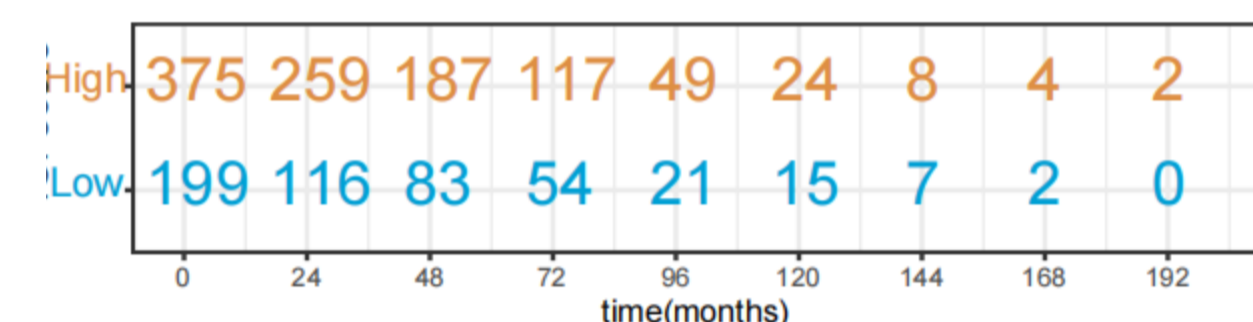

F

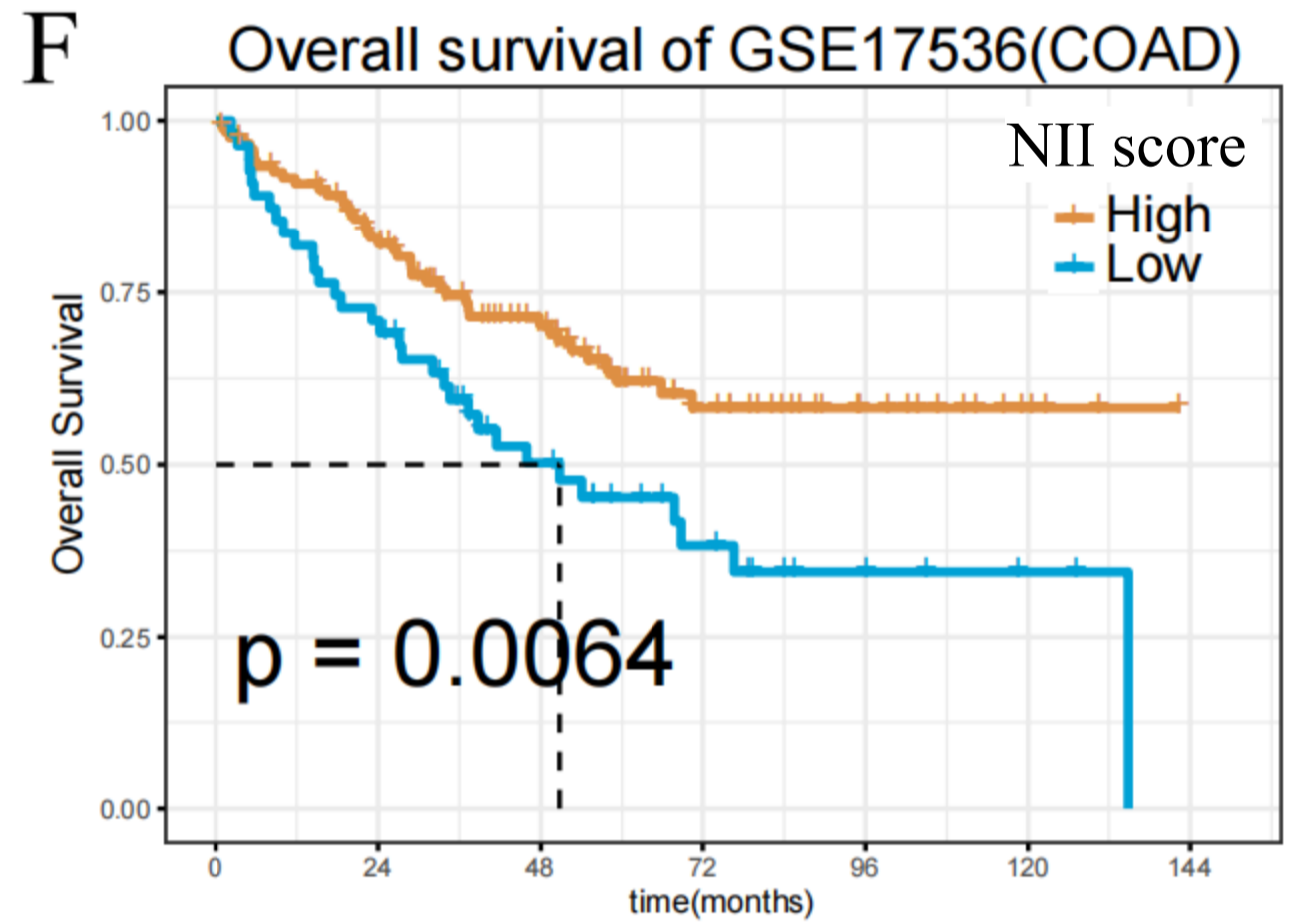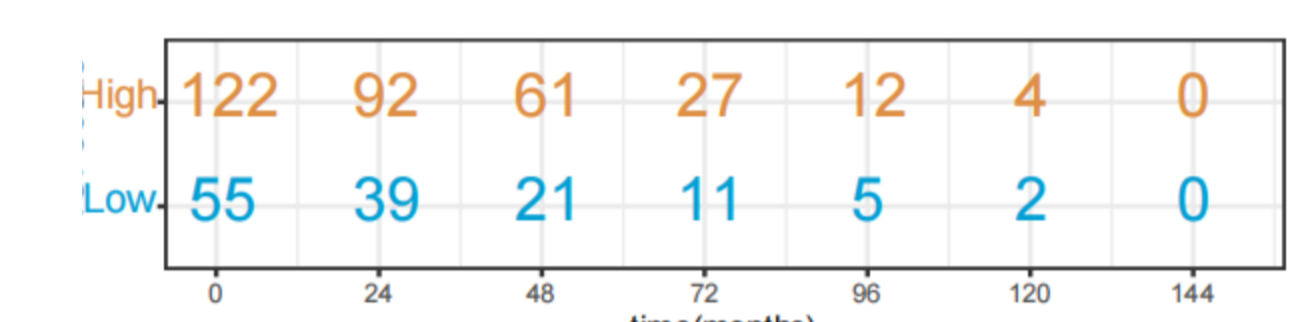

G

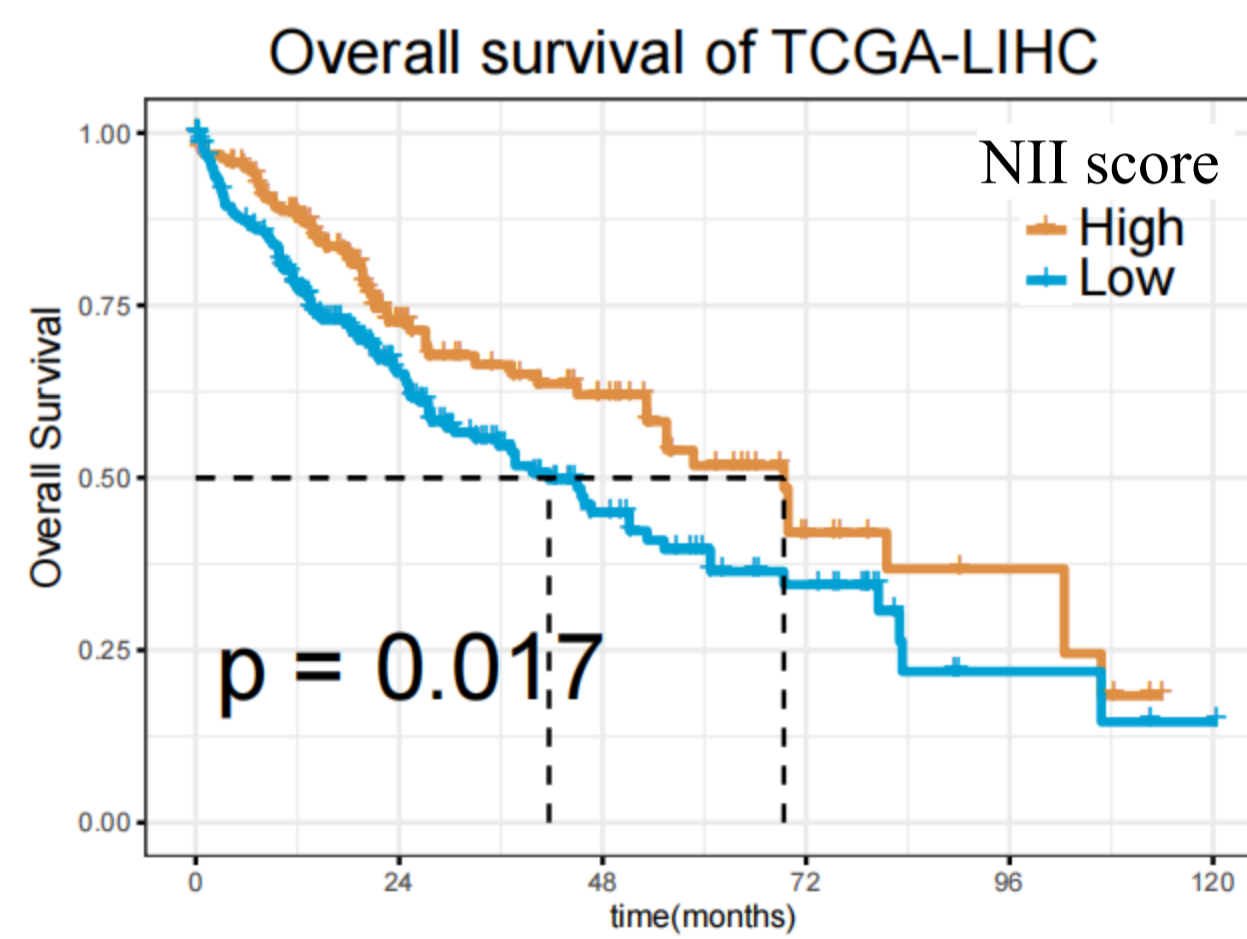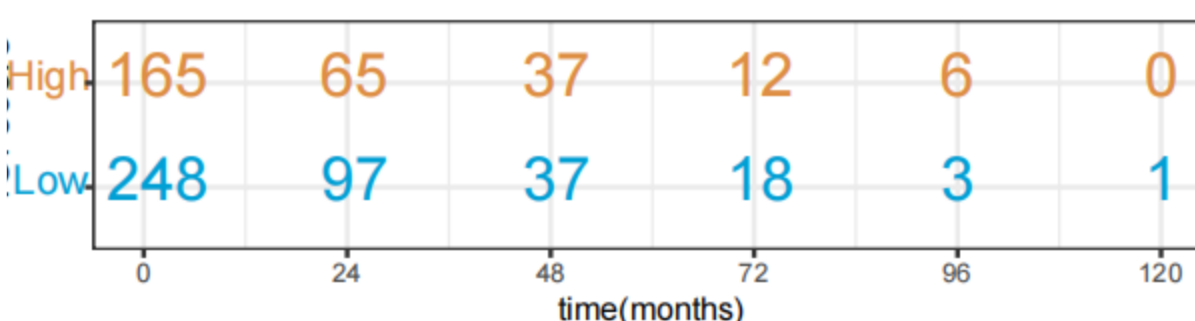

H

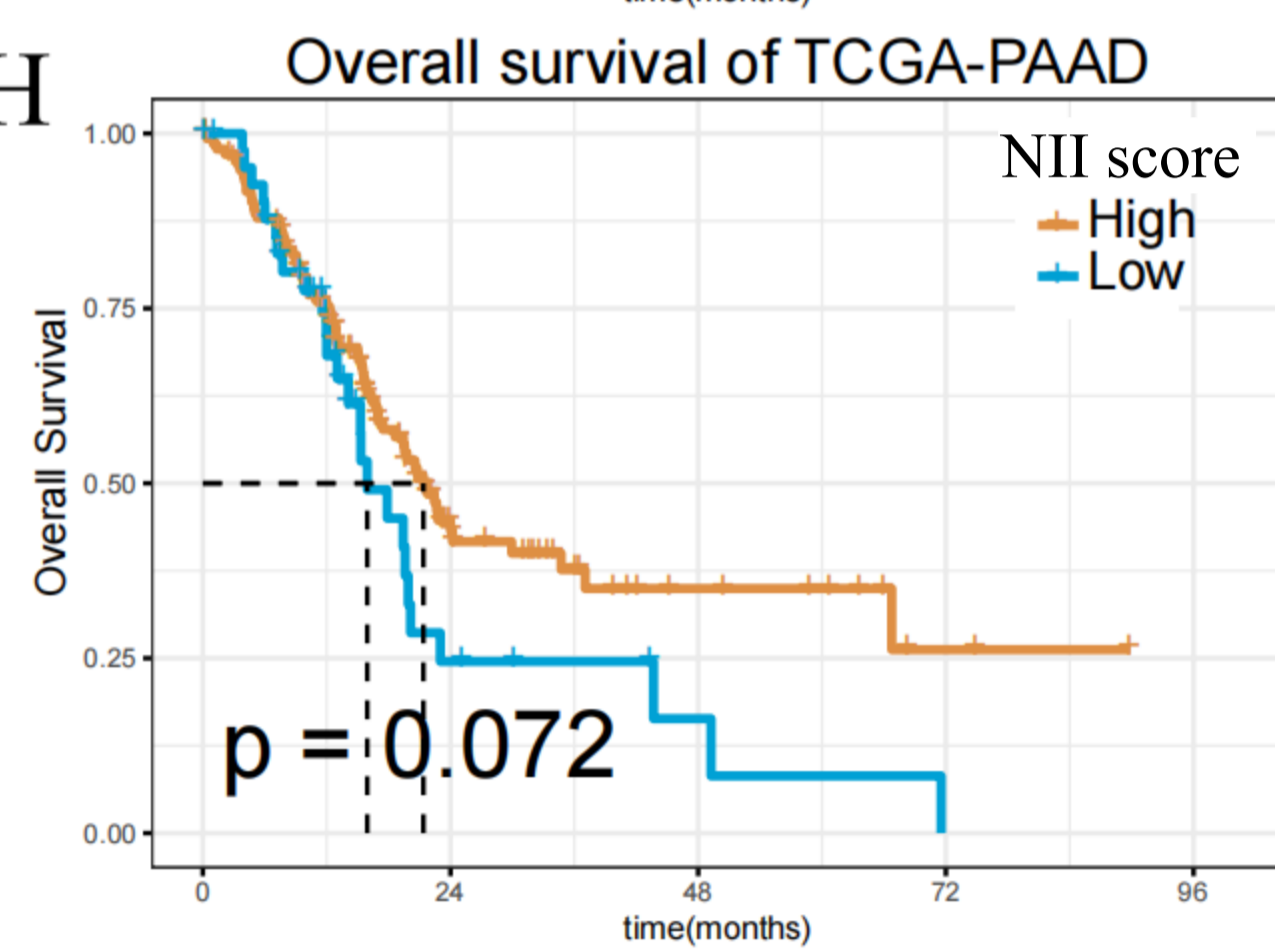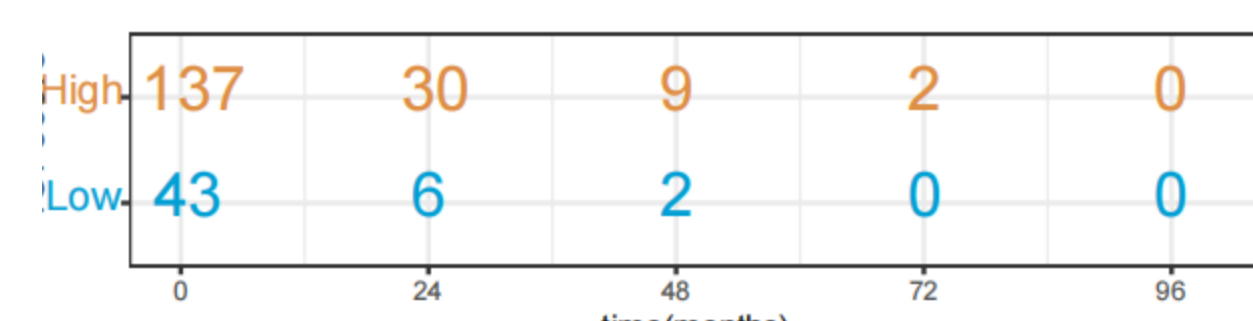

I

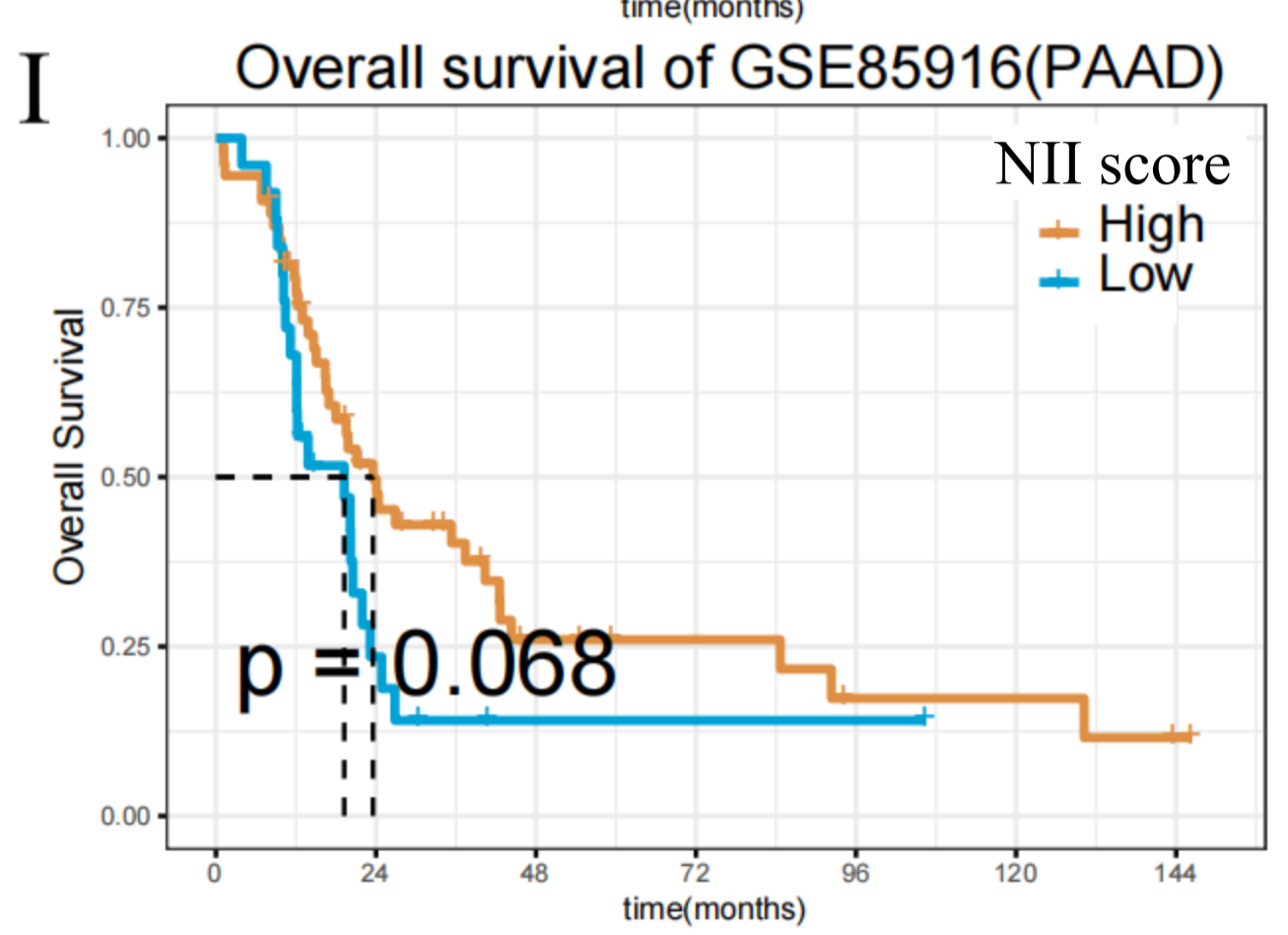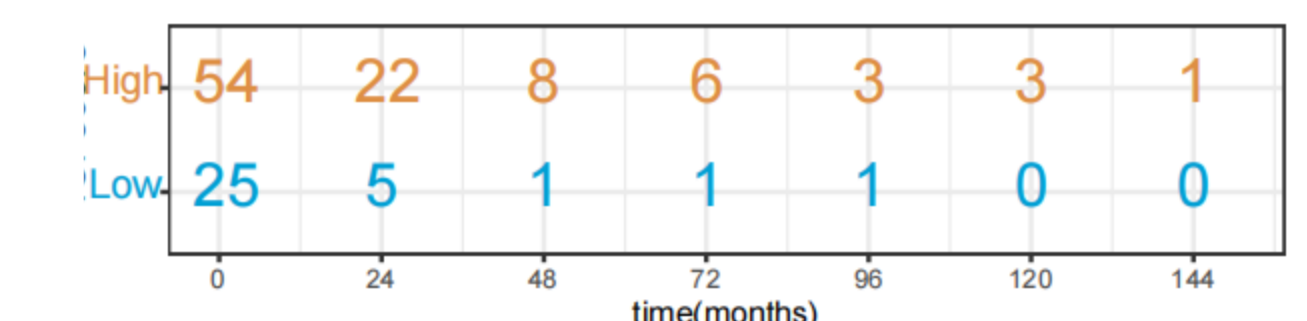

Supplement: Supplementary file 2 — Additional file 2: Figure S1. Establishment process and distribution of inflammation score. Figure S2. Establishment, prognostic significance and distribution of NII.cluster subtypes. Figure S3. The noteworthy differentially-expressed genes among NII cluster subtypes. Figure S4. The analyse of NII cluster subtypes and non-PNI in mutational signatures, methylation and CNV. Figure S5. Verification for prognosis of NII score in pan digestive tract cohorts. Figure S6. The verification that NII score serves as an independent prognostic factor using forest plots and subgroup analyses. Figure S7. The immune scores, stromal scores, ESTIMATE scores and potential therapeutic chemicals of NII score subgroups. Figure S8. The supplementary immunotherapy analyses of NII clusters and non-PNI in NII score subgroups. Figure S9. The result of chemosensitivity analyses. Figure S10. VCAM1 RNA expression of shRNA and overexpression cell models. [file 13046_2023_2730_MOESM2_ESM.zip › Figure S5.pdf]

FIGURE.S7

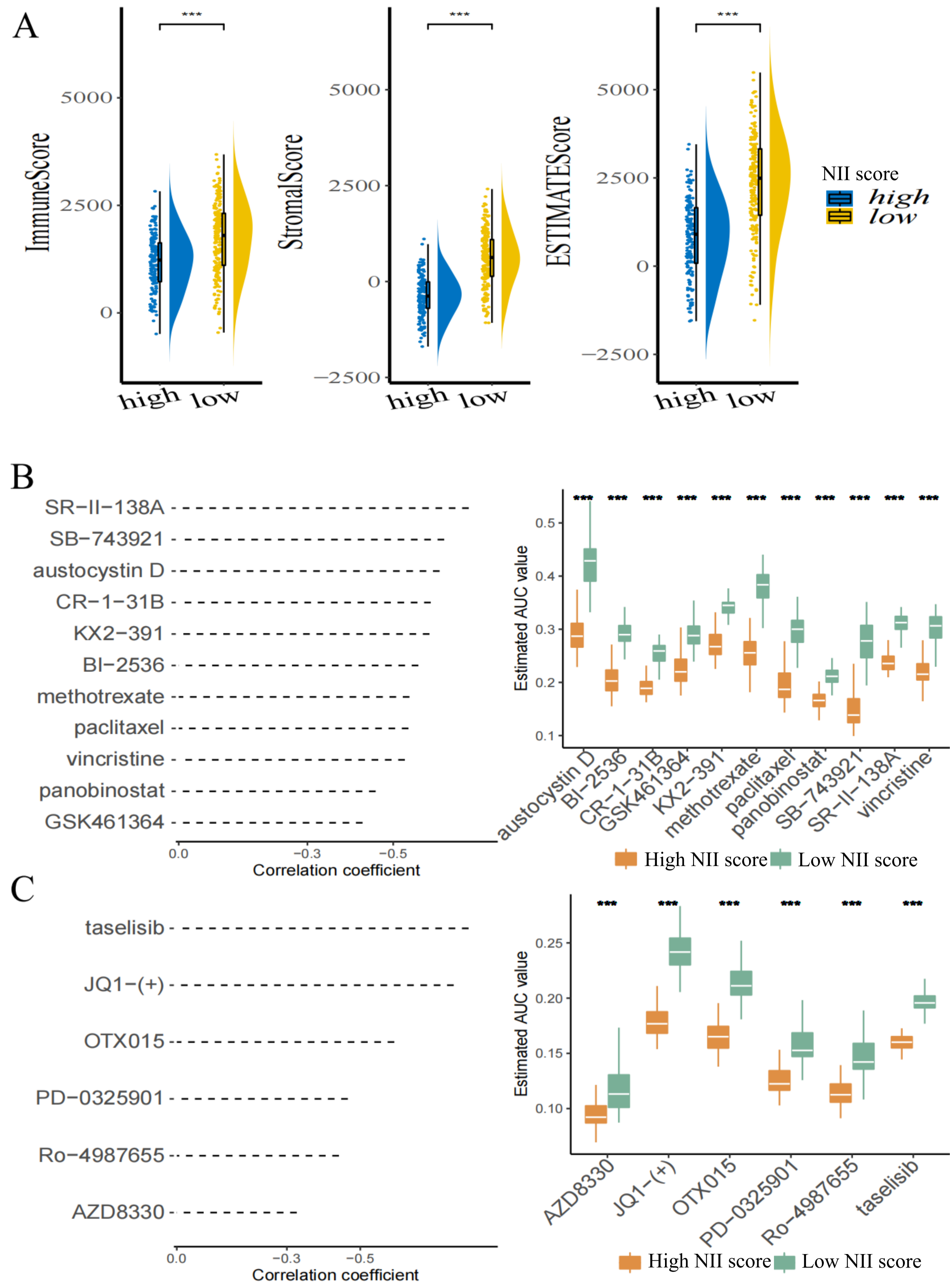

Supplement: Supplementary file 2 — Additional file 2: Figure S1. Establishment process and distribution of inflammation score. Figure S2. Establishment, prognostic significance and distribution of NII.cluster subtypes. Figure S3. The noteworthy differentially-expressed genes among NII cluster subtypes. Figure S4. The analyse of NII cluster subtypes and non-PNI in mutational signatures, methylation and CNV. Figure S5. Verification for prognosis of NII score in pan digestive tract cohorts. Figure S6. The verification that NII score serves as an independent prognostic factor using forest plots and subgroup analyses. Figure S7. The immune scores, stromal scores, ESTIMATE scores and potential therapeutic chemicals of NII score subgroups. Figure S8. The supplementary immunotherapy analyses of NII clusters and non-PNI in NII score subgroups. Figure S9. The result of chemosensitivity analyses. Figure S10. VCAM1 RNA expression of shRNA and overexpression cell models. [file 13046_2023_2730_MOESM2_ESM.zip › Figure S7.pdf]

FIGURE.S8

A

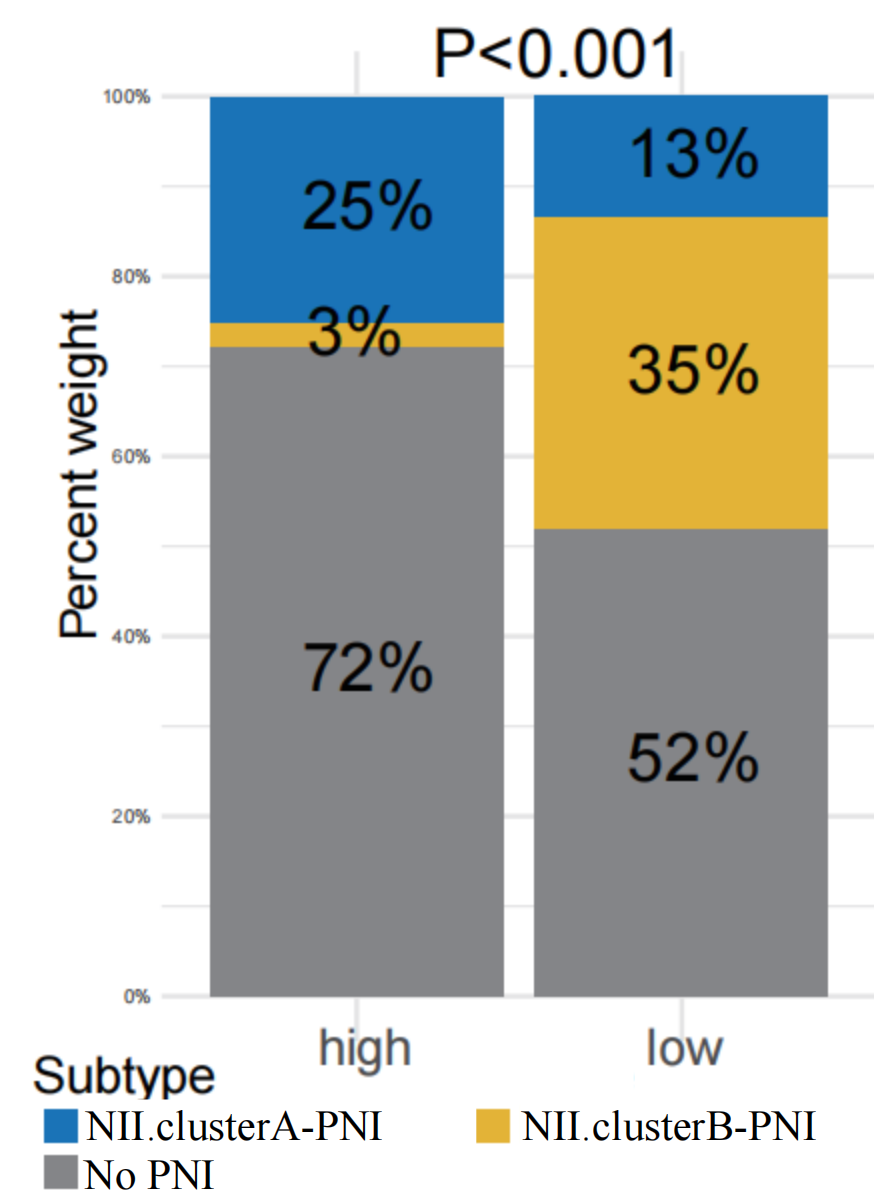

B

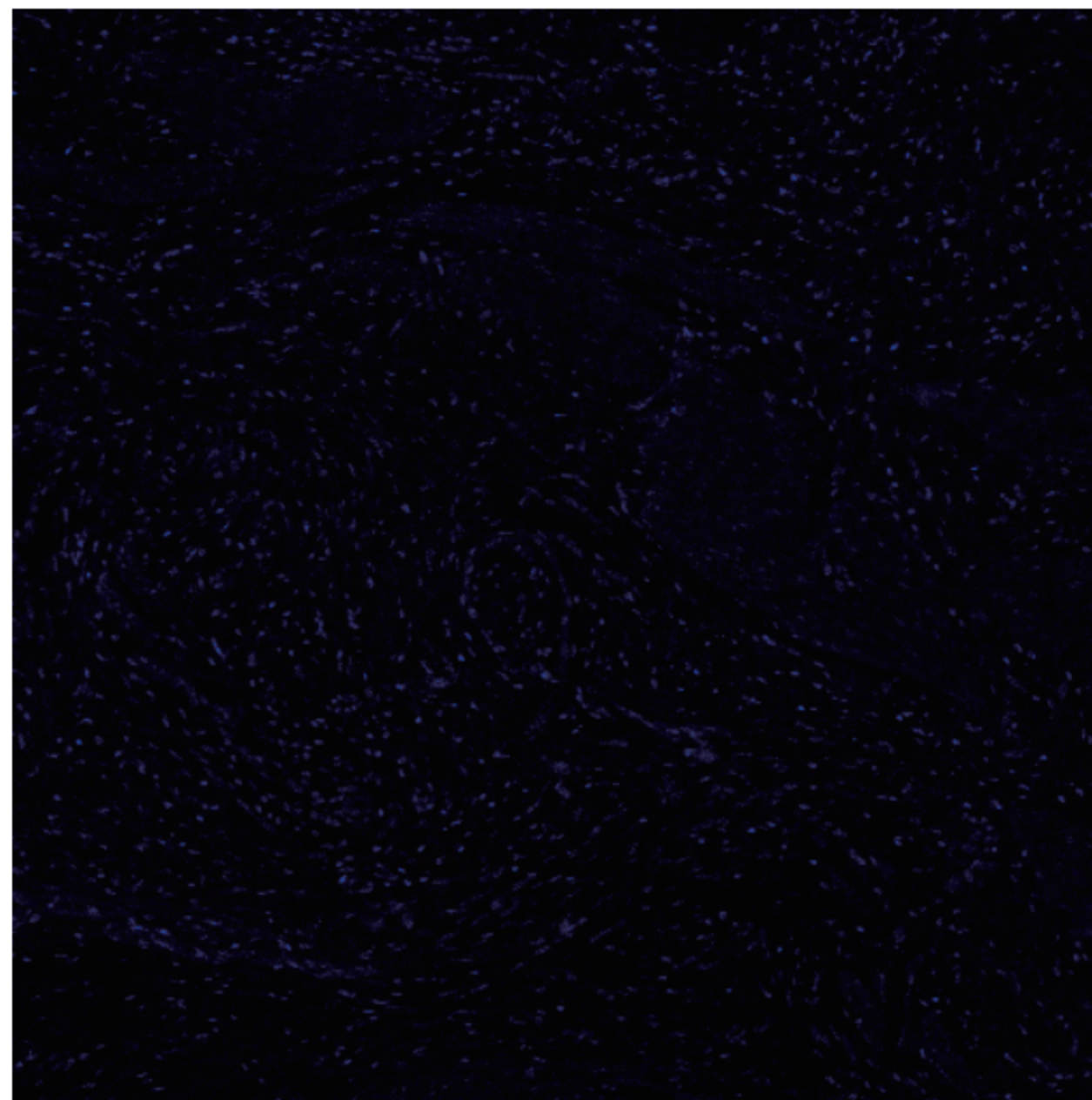

C

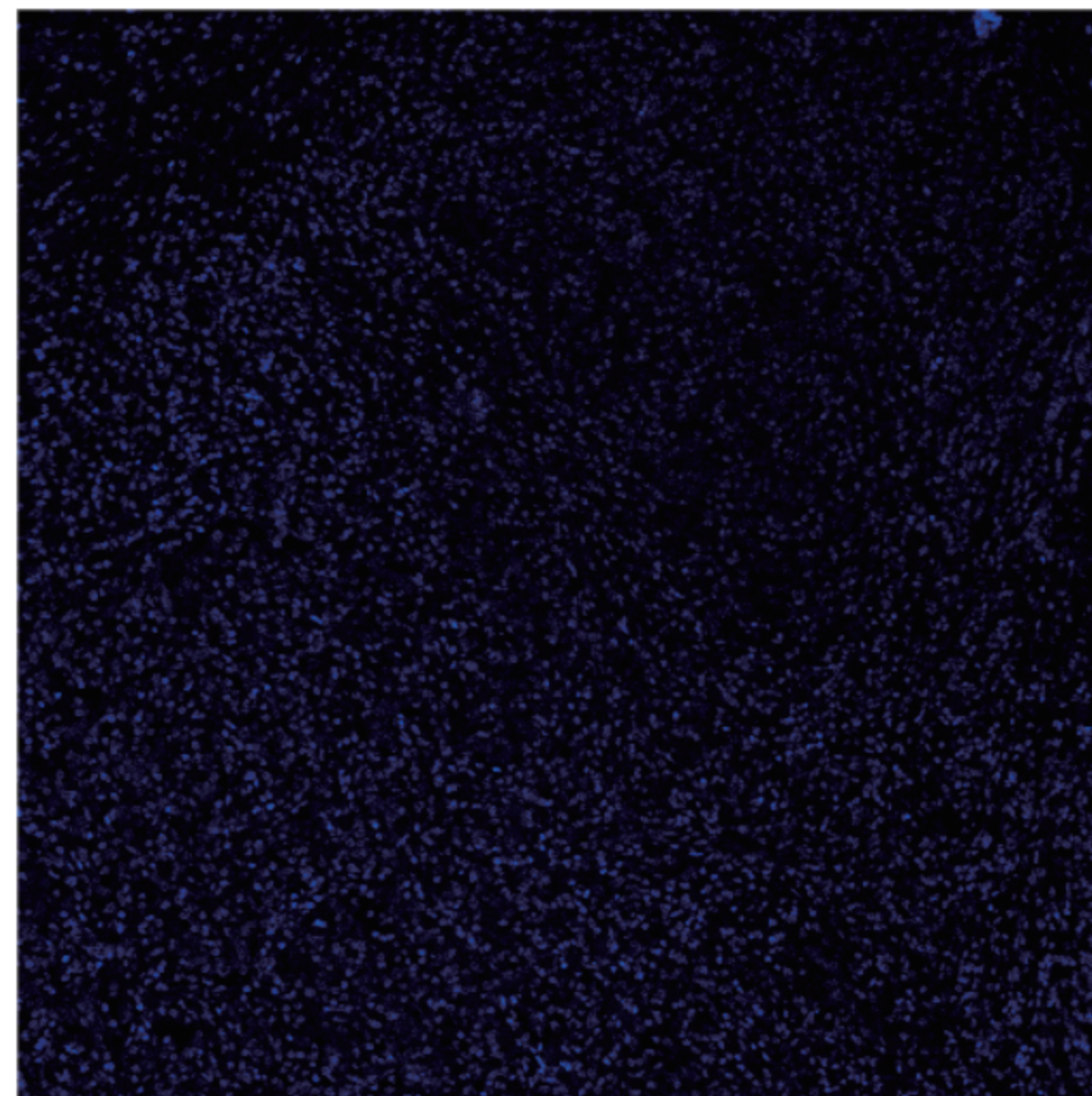

D

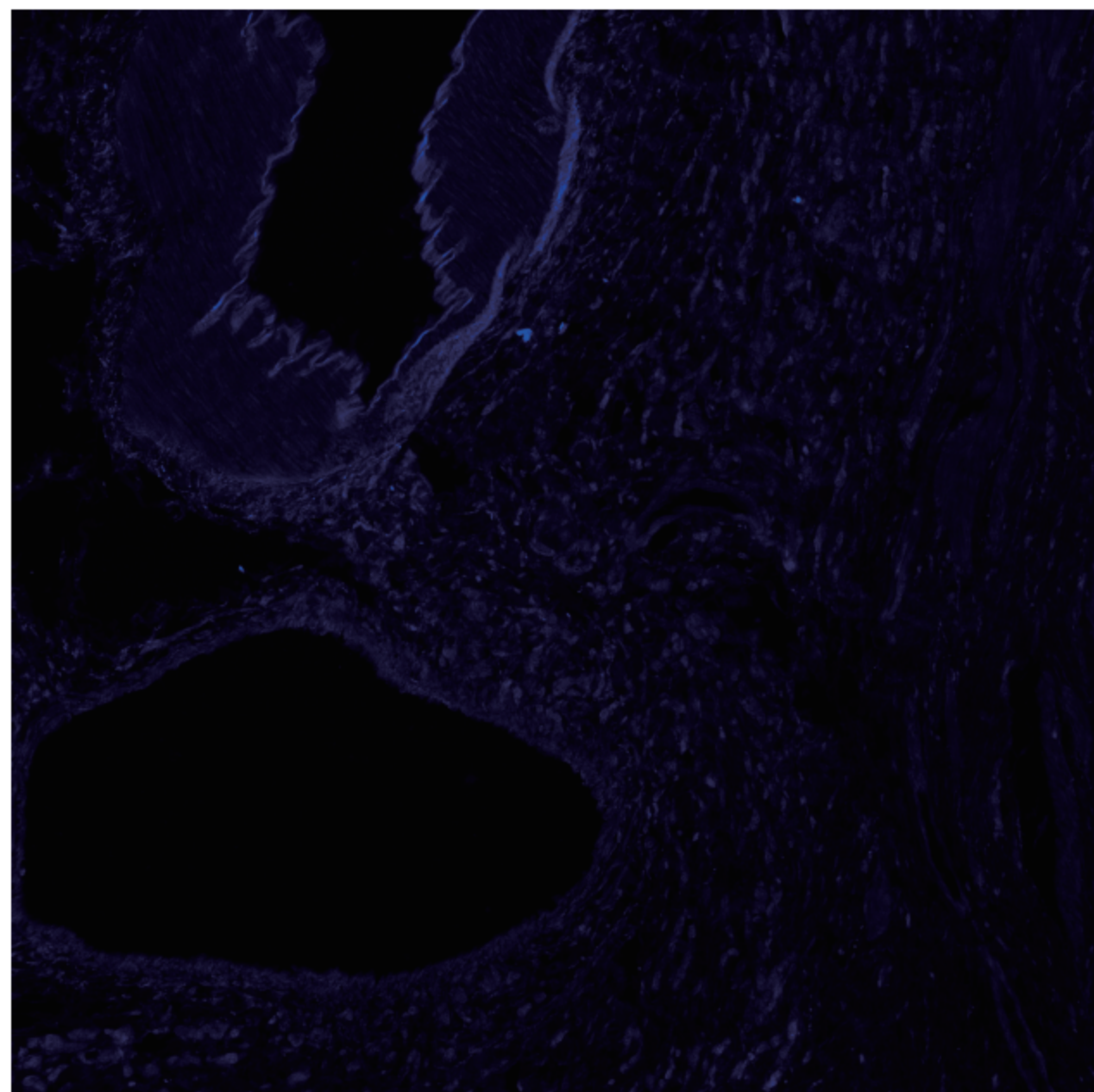

E

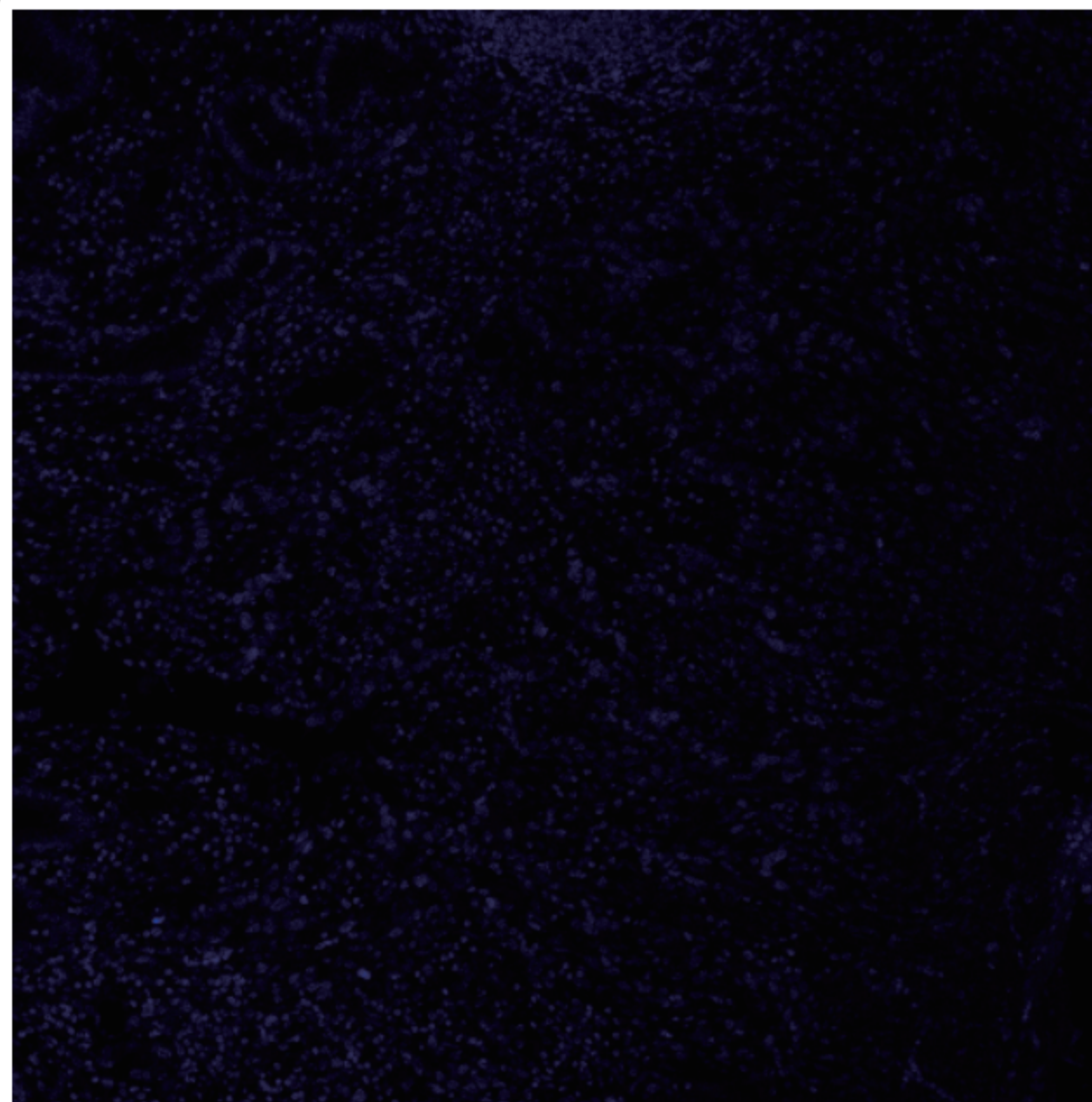

Supplement: Supplementary file 2 — Additional file 2: Figure S1. Establishment process and distribution of inflammation score. Figure S2. Establishment, prognostic significance and distribution of NII.cluster subtypes. Figure S3. The noteworthy differentially-expressed genes among NII cluster subtypes. Figure S4. The analyse of NII cluster subtypes and non-PNI in mutational signatures, methylation and CNV. Figure S5. Verification for prognosis of NII score in pan digestive tract cohorts. Figure S6. The verification that NII score serves as an independent prognostic factor using forest plots and subgroup analyses. Figure S7. The immune scores, stromal scores, ESTIMATE scores and potential therapeutic chemicals of NII score subgroups. Figure S8. The supplementary immunotherapy analyses of NII clusters and non-PNI in NII score subgroups. Figure S9. The result of chemosensitivity analyses. Figure S10. VCAM1 RNA expression of shRNA and overexpression cell models. [file 13046_2023_2730_MOESM2_ESM.zip › Figure S8.pdf]

FIGURE.S9

A

CTPR

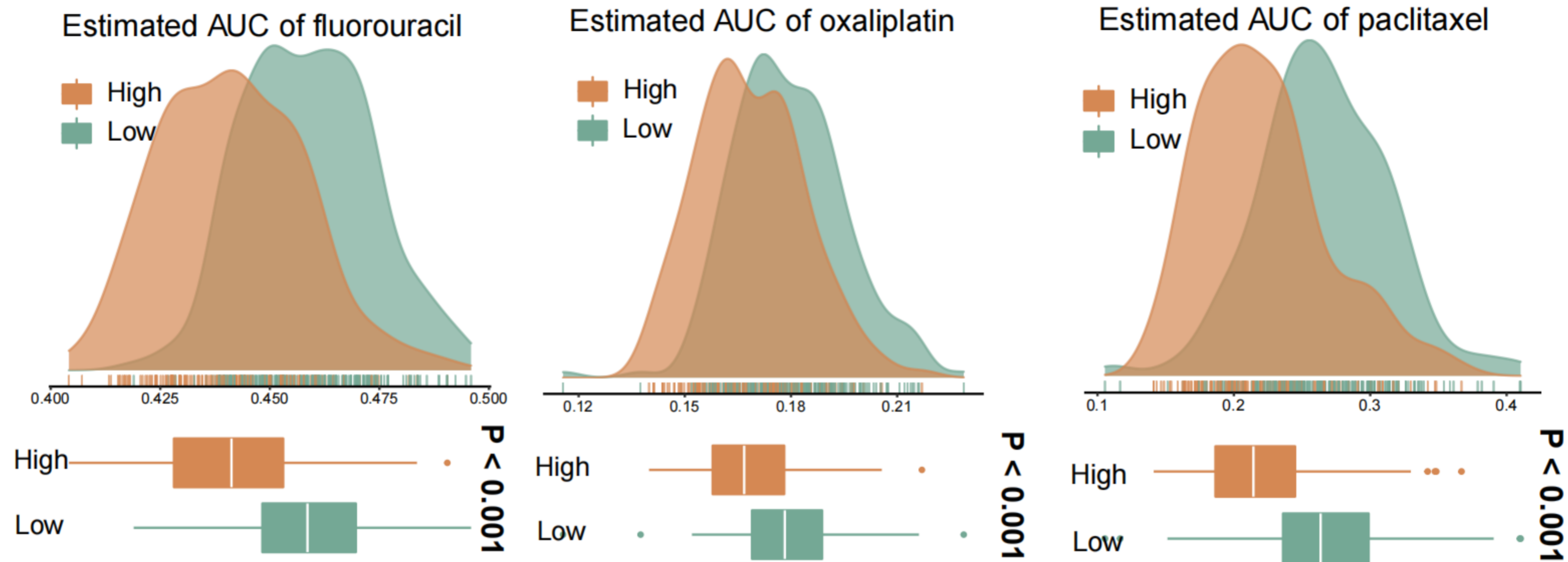

B

PRISM

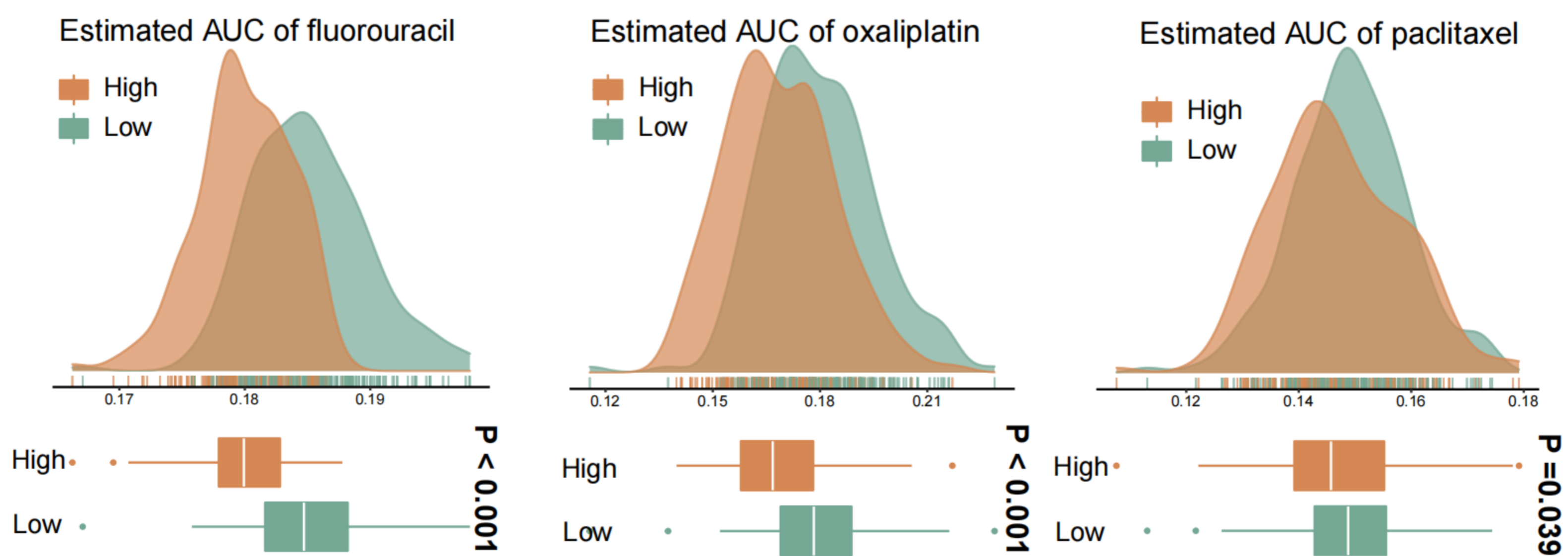

C

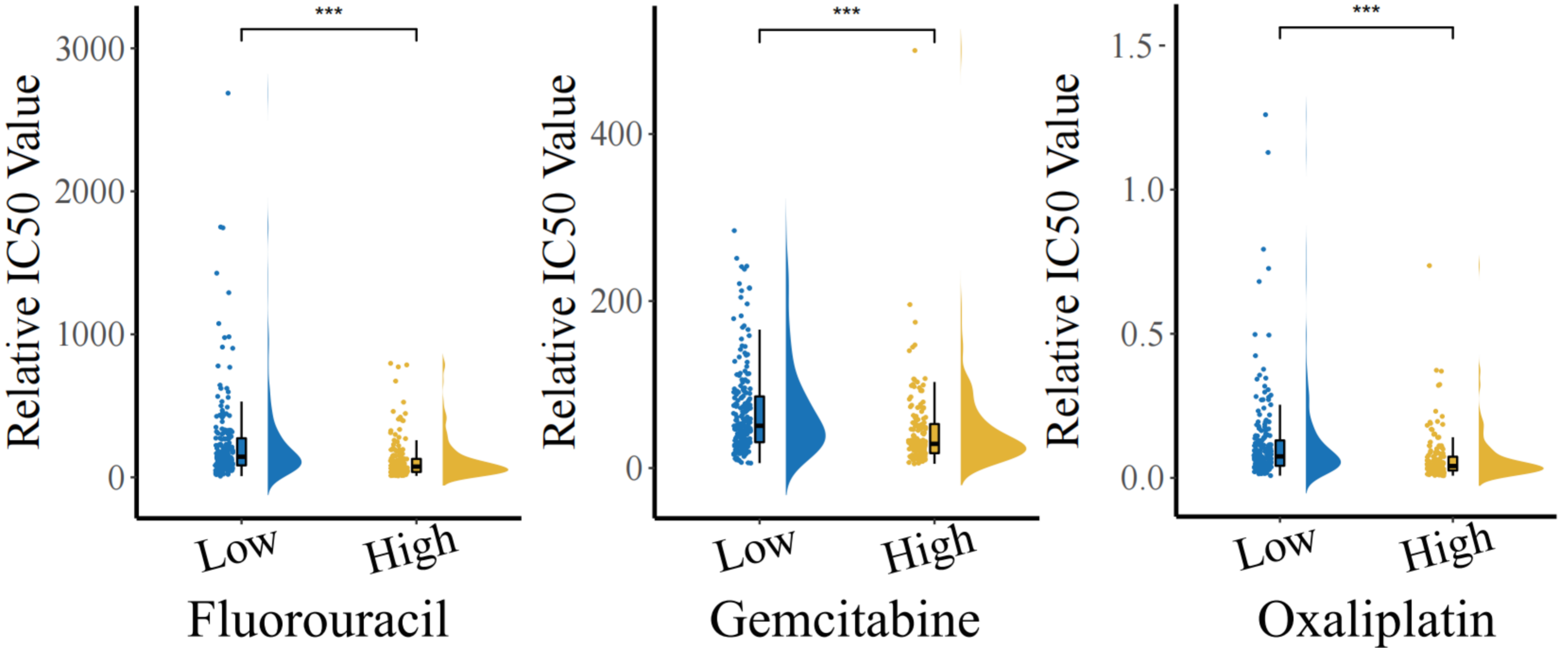

NII score 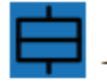 Low 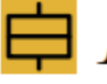 High

Supplement: Supplementary file 2 — Additional file 2: Figure S1. Establishment process and distribution of inflammation score. Figure S2. Establishment, prognostic significance and distribution of NII.cluster subtypes. Figure S3. The noteworthy differentially-expressed genes among NII cluster subtypes. Figure S4. The analyse of NII cluster subtypes and non-PNI in mutational signatures, methylation and CNV. Figure S5. Verification for prognosis of NII score in pan digestive tract cohorts. Figure S6. The verification that NII score serves as an independent prognostic factor using forest plots and subgroup analyses. Figure S7. The immune scores, stromal scores, ESTIMATE scores and potential therapeutic chemicals of NII score subgroups. Figure S8. The supplementary immunotherapy analyses of NII clusters and non-PNI in NII score subgroups. Figure S9. The result of chemosensitivity analyses. Figure S10. VCAM1 RNA expression of shRNA and overexpression cell models. [file 13046_2023_2730_MOESM2_ESM.zip › Figure S9.pdf]
